# Mapping the early life gut microbiome in neonates with critical congenital heart disease: multiomics insights and implications for host metabolic and immunological health

**DOI:** 10.1186/s40168-022-01437-2

**Published:** 2022-12-30

**Authors:** Yuan Huang, Wenlong Lu, Min Zeng, Xiaoyue Hu, Zhanhao Su, Yiwei Liu, Zeye Liu, Jianhui Yuan, Li Li, Xiaoling Zhang, Long Huang, Wanjin Hu, Xu Wang, Shoujun Li, Hao Zhang

**Affiliations:** 1 State Key Laboratory of Cardiovascular Disease, Fuwai Hospital, National Center for Cardiovascular Diseases, Pediatric Cardiac Surgery Center, Fuwai Hospital, Chinese Academy of Medical Sciences, and Peking Union Medical College, Beijing, China; 2 PICU, Pediatric Cardiac Center, Fuwai Hospital, Chinese Academy of Medical Science and Peking Union Medical College, Beijing, China; 3 Department of Neonatology, Affiliated Children’s Hospital of Capital Institute of Pediatrics, Beijing, China; 4 Heart Center and Shanghai Institute of Pediatric Congenital Heart Disease, Shanghai Children’s Medical Center, National Children’s Medical Center, Shanghai Jiaotong University School of Medicine, Shanghai, China; 5 Shanghai Majorbio Bio-Pharm Technology Co, Shanghai, China

**Keywords:** Intestinal microbiology, Congenital heart disease, Metabolic and immune homeostasis, Clinical prognosis

## Abstract

**Background:**

The early life gut microbiome is crucial in maintaining host metabolic and immune homeostasis. Though neonates with critical congenital heart disease (CCHD) are at substantial risks of malnutrition and immune imbalance, the microbial links to CCHD pathophysiology remain poorly understood. In this study, we aimed to investigate the gut microbiome in neonates with CCHD in association with metabolomic traits. Moreover, we explored the clinical implications of the host-microbe interactions in CCHD.

**Methods:**

Deep metagenomic sequencing and metabolomic profiling of paired fecal samples from 45 neonates with CCHD and 50 healthy controls were performed. The characteristics of gut microbiome were investigated in three dimensions (microbial abundance, functionality, and genetic variation). An in-depth analysis of gut virome was conducted to elucidate the ecological interaction between gut viral and bacterial communities. Correlations between multilevel microbial features and fecal metabolites were determined using integrated association analysis. Finally, we conducted a subgroup analysis to examine whether the interactions between gut microbiota and metabolites could mediate inflammatory responses and poor surgical prognosis.

**Results:**

Gut microbiota dysbiosis was observed in neonates with CCHD, characterized by the depletion of *Bifidobacterium* and overgrowth of *Enterococcus*, which was highly correlated with metabolomic perturbations. Genetic variations of *Bifidobacterium* and *Enterococcus* orchestrate the metabolomic perturbations in CCHD. A temperate core virome represented by *Siphoviridae* was identified to be implicated in shaping the gut bacterial composition by modifying microbial adaptation. The overgrowth of *Enterococcus* was correlated with systemic inflammation and poor surgical prognosis in subgroup analysis. Mediation analysis indicated that the overgrowth of *Enterococcus* could mediate gut barrier impairment and inflammatory responses in CCHD.

**Conclusions:**

We demonstrate for the first time that an aberrant gut microbiome associated with metabolomic perturbations is implicated in immune imbalance and adverse clinical outcomes in neonates with CCHD. Our data support the importance of reconstituting optimal gut microbiome in maintaining host metabolic and immunological homeostasis in CCHD.

Video Abstract

**Supplementary information:**

The online version contains supplementary material available at 10.1186/s40168-022-01437-2.

## Introduction

The early life gut microbiome is a crucial modulator in the health and development of the host [1]. Mounting evidence suggests that multilevel microbial traits that underpin host–microbiome crosstalk during infancy have far-reaching effects on host metabolic and immunological homeostasis, such as facilitating nutrient acquisition, promoting immune maturation and tolerance development [2, 3], enabling pathogen clearance [4], and nourishing intestinal epithelial cells [5]. By contrast, the absence or disruption of optimal host–microbiome interactions during this critical window of development would therefore be expected to have detrimental effects on specific functions or potentially on regulation of the host system as a whole. From this perspective, deciphering the clinical implications of the early life gut microbiome is of critical importance in guiding health promotion and disease prevention.

In the past decade, rapid developments in deep shotgun sequencing and metagenome-wide bioinformatics have enabled in-depth characterization of the infant gut microbiome and have unraveled the complex microbial links to host pathophysiology in multiple clinical scenarios, such as prematurity and food allergy [6–8]. However, little is known about the impact of the early life gut microbiome in patients with congenital heart disease (CHD), despite CHD being the most common birth defect—affecting more than three million neonates worldwide in 2019 [9]. Indeed, nearly 25% of CHD cases are critical (CCHD) [10, 11], which remains an important cause of infant mortality under the age of 1 year. Although advancements in surgical techniques and perioperative management have substantially reduced mortality for the entire spectrum of CHD, the occurrence of periprocedural complications in CCHD remains a significant risk of death, with infection, excessive inflammatory response, and malnutrition being the major extracardiac risks contributing to poor clinical outcomes [12–14]. Notably, up to 50% of neonates with CCHD require cardiac surgery within the first month of life [15], and the immune and nutritional status prior to surgery are highly correlated with prognosis [13, 14]. In view of long-lasting hyoxemia and abnormal gut perfusion secondary to poor cardiac output in neonates with CCHD, gut dysbiosis and epithelial barrier impairment are postulated to be implicated in metabolic disorders and inflammatory cascades. In addition, our previous research on cyanotic CHD indicated that chronic hypoxia could induce alterations in the gut microbiome and predispose bone marrow mesenchymal stem cells to premature senescence [16], further implying the role of aberrant gut microbiome in mediating immune dysfunction in the context of CHD pathology. Notwithstanding this plausible evidence, it remains unclear whether CCHD is accompanied by enteric microbial disturbances, and the extent to which the early life gut microbiome is implicated in CCHD pathology remains to be fully investigated.

In this study, we performed an integrated analysis of fecal metabolomics and metagenome sequencing of paired samples from neonates with CCHD and healthy controls (HCs). We aimed to (i) systematically characterize the early life gut microbiome in neonates with CCHD in three dimensions (microbial composition, functionality, and genetic variation) and uncover its implications on the host metabolic phenotype; (ii) reveal the ecological interaction between the gut viral and bacterial communities and unravel its impacts on the overall microbial configuration. To gain further biological insights, we profiled serum levels of proinflammatory cytokines and biomarkers of intestinal permeability in the patient cohort, and used metagenome-wide association analysis and bi-directional mediation analysis to infer the potential regulatory relationships among gut microbiota, epithelial barrier impairment, inflammatory response, and surgical prognosis.

## Material and methods

The detailed information about study design, settings, and methods of bioinformatic analyses is available in Additional file 1.

### Participants’ recruitment and samples collection

This study was approved by the Human Research and Ethics Committee of Fuwai Hospital Chinese Academy of Medical Sciences (Approval No. 2019–1300) and Shanghai Children’s Medical Center (Approval NO. SCMCIRB-K2021021). Full-term neonates with CCHD [17] admitted to the Pediatric Cardiac Surgery Center from December 2019 to August 2021 were enrolled. The exclusion criteria included (i) diagnosed with any extra-cardiac medical condition that could affect metabolism, nutritional status or physical health; (ii) previous usage of probiotics, antibiotics or immunosuppressant which could potentially affect gut microbial composition. The enrollment flowchart is shown in Additional file 1: Figure S1. A total of 45 neonates with CCHD were finally included. Fifty matched healthy controls were recruited simultaneously. Informed consent was obtained from all the guardians of the participants for samples and data collection. Fecal samples were scrapped from diapers and immediately frozen at – 80 °C until analysis. Blood samples were collected on admission from CCHD patients and preprocessed to obtain serum (centrifuged at 3000 rpm for 10 min and collected the supernatant), and then stored at – 80 ℃ until analysis. The baseline demographics and clinical metadata were collected from electronic medical records.

### Metagenomic sequencing

Briefly, genomic DNA was isolated from fecal samples using QIAamp PowerFecal Pro DNA Kit (QIAGEN, USA) according to the manufacturer’s instructions. Concentration and purity of extracted DNA were confirmed by TBS-380 and NanoDrop2000, respectively. Paired-end library was constructed using NEXTFLEX Rapid DNA-Seq (Bioo Scientific, USA). Sequencing was performed on a Novaseq 6000 platform (Illumina, USA) and generated a sequencing depth of approximately 117 million raw reads per sample. Fastp (https://github.com/OpenGene/fastp, version 0.20.0) was utilized to remove the adaptors and low-quality reads. Host reads were removed after alignment with the human genome assembly (GRCh38.p13) using Burrows-Wheeler Aligner (http://bio-bwa.sourceforge.net). The high-quality non-host reads were assembled using MEGAHIT (https://github.com/voutcn/megahit, version 1.1.2) and contigs with lengths greater than 300 bp were used for gene (ORFs) prediction by Prodigal (version 2.6.3) [18]. Only ORF longer than 100 bp was considered in downstream analyses. Gene sequences were clustered into a non-redundant gene catalogue using CD-HIT (version 4.6) [19] at 95% identity and 90% coverage. The high-quality reads were mapped to the non-redundant gene catalogue with 95% identity using SOAPaligner (http://soap.genomics.org.cn/, version 2.21).

Bacteria taxonomic and functional profiles were obtained with the resulted gene sets aligned to the NCBI NR database and KEGG database (http://www.genome.jp/kegg/) using Diamond (version 2.0.11) at the *e* value = 1e − 5. The microbiome diversity analyses (including alpha and beta diversity) were conducted and visualized using the vegan and ggplot2 packages in R (version 4.0.2). The discriminative bacterial species and KEGG orthology between the two groups were identified using LEfSe with a LDA score > 2.0.

### Untargeted LC–MS–based metabolomics

Untargeted metabolomics analysis was conducted as previously described [20]. Briefly, metabolic extracts were obtained from fecal samples following methanol-assisted protein precipitation and then analyzed by an UHPLC system (Vanquish, Thermo Fisher Scientific) with a UPLC BEH Amide column. The raw data were converted to the mzXML format using ProteoWizard and processed with R package xcms [21], for peak detection, extraction, alignment, and integration. The in-house MS2 database was applied for metabolite identification. The OPLS-DA was performed using the statistics function prcomp in R (version 4.0.2). In order to avoid overfitting, a permutation test (200 permutations) was performed. The discriminative metabolites between the two groups were identified with variable importance in the projection (VIP) > 1 (determined by OPLS-DA) and *P* < 0.05 (determined by Student’s t test). The KEGG database (http://www.genome.jp/kegg/) and MetaboAnalyst database (http://www.metaboanalyst.ca/) were used for pathway enrichment analysis.

### Targeted metabolites measurement of SCFAs

The SCFA concentrations were determined by gas chromatography-mass spectrometry. The detailed methodology is available in Supplementary file.

### Analysis of serum proinflammatory cytokines and intestinal permeability biomarkers

The Luminex Human Discovery multiplex assay kit (Catalog Number: LXSAHM; R&D Systems, Inc, USA) was used for simultaneous measurement of cytokines IL-1β, IL-6, IL-8, TNF-α, and INF-γ in serum samples. Upon completion of each multiplex assay, the amounts of serum cytokines were analyzed via Luminex. The commercially available ELISA kits (Bio-swamp Life Science, Wuhan, China) were used to measure the intestinal permeability biomarkers according to manufactures’ instructions, including zonulin, D-lactate, iFABP, LPS, and LBP.

### Characterization of microbial genetic structural variations

SGV-Finder pipeline [22] was applied to classify the microbial structural variations (SVs). The SV-screening procedure is mainly divided into two steps: (1) run ICRA, which is an ‘iterative coverage-based read assignment’ algorithm, to resolve ambiguous read assignments to regions that are similar between different bacteria. (2) run SGV-Finder, which analyses coverage depth across all microbial genomes in all samples to characterize SVs with respect to the standardized coverage of a genome in a given sample (https://doi.org/10.1038/s41586-019-1065-y). The variability of microbial SVs between individuals were determined by Canberra distance.

### Virome analysis based on metagenomic sequencing data

1. ***Identification of viral contigs***
  The putative viral contigs were identified from the bulk metagenomes using DeepVirFinder [23] with default settings. Only predicted contigs with lengths greater than 1000 bp were extracted, and those with P value < 0.05 and score ≥ 0.7 were considered as candidate virus contigs for subsequent analysis.
2. ***Viral taxonomic profiling***
  To ensure the accuracy of viral taxonomic annotation, five latest improved databases were employed to classify taxonomy of putative viral contigs: (1) viral RefSeq genomes database (virus reference genome sequences downloaded from NCBI) (https://www.ncbi.nlm.nih.gov/labs/virus/vssi/#/virus?SeqType_s=Genome); (2) Gut Phage Database (GPD) [24]. (3) Metagenomic Gut Virus (MGV) catalogue [25]. (4) IMG/VR database (version: IMG_VR_2020-10-12_5.1-IMG/VR v3) [26]. (5) Virus Pathogen Resource (ViPR, https://www.viprbrc.org/brc/home.spg?decorator=vipr) [27]. Technically, the viral contigs were successively aligned to the above-mentioned databases using DIAMOND with an *E* value < 10^−5^, and select the database alignment result with the highest identity and the most complete virus classification information as the final virus taxonomic information. A total of 22,245 virus contigs were successfully annotated and used for subsequent analyses.
3. *Prediction of phage lifestyles*
  PHACTS was used to classify phage lifestyles (i.e., temperate or lytic bacteriophages) of putative viral contigs. In specific, of 22,245 viral contigs, 22,174 were classified as “Bacteriophages” and used for PHACTS analysis. Ten replicate PHACTS predictions were performed. Probability values obtained from PHACTS were standardized between − 1 and 1, which was presented as probability of “Lytic” or “Temperate”.

### Prophage-based bacteria-phage association analysis

Prophage-based bacteria-phage association analysis was conducted as previously described [28, 29]. In brief, the prophage sequences in bacterial contigs were predicted using VirSorter (version 1.0.3) [30]. Initially, the bacterial contigs (≥ 5 kb) were analyzed by VirSorter using both RefSeqABVir (− db 1) and Viromes (− db 2). Then the predicted prophage sequences of VirSorter categories 4 or 5 (presence of viral hallmark genes or enrichment of viral-like genes in a prophage region) were extracted. The positions of the predicted prophage sequences on bacterial contigs were determined through megablast (BLAST + version 2.7.1) searches (*E* value < 10^−100^ and ≥ 95% identity), and prophage sequences were merged if their positions overlapped. Prophage sequences longer than 3 kb were extracted and listed as final prophage sequences.

Gene annotation of viral contigs was based on open reading frames (ORFs). The ORFs on the viral contigs were predicted by MetaProdigal (version 2.6.3) [31]. The identified ORFs were then queried by hmmscan in HMMER3 (version 3.3.2) [32] against the PfamA database (version 34.0) [33], VFDB [34], and CARD [35] with an *E* value < 10^−5^.

### CRISPR-based bacteria-phage association analysis

The CRISPR-based bacteria-phage association analysis was conducted as previously described [28, 29]. Briefly, we first identified CRISPR spacers on bacterial contigs (≥ 5 kb) using CRISPRDetect [36]. To identify the target phages of the CRISPR spacers, we then queried the identified spacers using blastn (BLAST + version 2.12.0) (https://doi.org/10.1186/1471-2105-10-421) against the viral contigs and extracted the aligned spacers with > 90% of their length aligned with a minimum identity level of 95% and a maximum *E* value of 5 × 10^−3^.

### Enterotype analysis

Enterotype analysis was performed at the genus level using the Jensen-Shannon divergence (JSD) distance and the Partitioning Around Medoids (PAM) clustering algorithm in the R package cluster. The optimal number of clusters was determined by Calinski-Harabasz (CH) index using the R package clusterSim. PCoA was performed to visualize the clusters of samples using the R package ade4 [37]. The top 10 most abundant species enriched in each enterotype were selected for Spearman’s rank correlation analysis. For plotting purpose, only correlations with coefficient >  + 0.6 or <  − 0.6 were plotted in the network using Cytoscape 3.9.0.

### Metagenome-wide association analysis

The significant associations between continuous microbial variables (microbial abundance, KEGG pathway abundance, microbial vSVs) and fecal metabolites were determined by Spearman’s rank correlation analysis. Two-sided Mann–Whitney *U* test was used to calculate significance of associations between binary variables (dSVs) and the same metabolites.

### Mediation analysis

To investigate the mediation linkages between continuous microbial features, fecal metabolites, and host pathological phenotypes (i.e., serum biomarkers of inflammation and intestinal permeability), we first identified microbe-metabolite-biomarker groups in which all variables significantly correlated with each other as candidate groups with a potential mediation effect. Then we performed bi-directional analysis on the candidate groups using the R package mediation (version 4.5.0).

### Statistical analysis

Basic statistical analyses (including Student’s *t* test, Wilcoxon test, chi-square test, and Fisher’s exact test) were performed as appropriate to compare continuous and categorical variables using SPSS (version 25). Univariable and multivariable logistic regression analyses were performed to evaluate the predictive value of *Enterococcus* abundance in prognostic stratification in the study cohort (SPSS version 25). Receiver-operating characteristic curve was used to evaluate the discriminative performance of the predictive model. Other statistical details related to metagenomic and metabolomic data can be found in the figure legends and methods. *P* values of < 0.05 were considered significant.

## Results

### Baseline characteristics of the participants

Forty-five full-term neonates diagnosed with CCHD and 50 HCs matched by age and gender were enrolled in this study. The enrollment flowchart and cohort characteristics are shown in Additional file 1: Figure S1 and Table S1, respectively. In brief, no significant differences were observed in birth weight, delivery mode, and breastfeeding status between the two groups.

### Differences in gut bacteria between CCHD patients and HCs

**Fig. 1:**
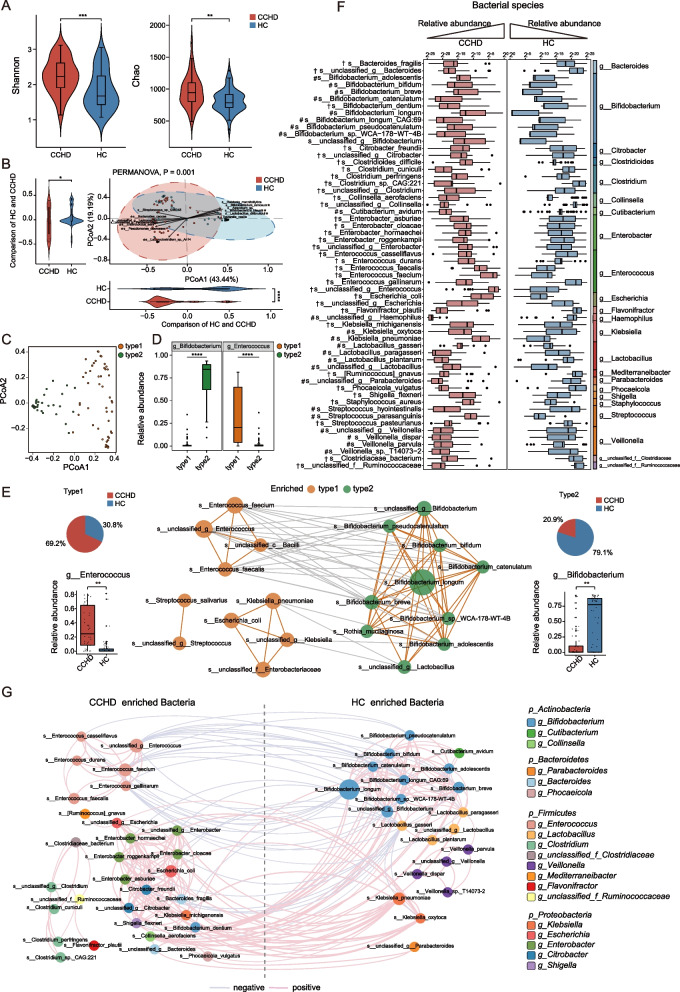
Gut bacterial characteristics in CCHD patients and HCs. **A** The comparison of gut bacterial α-diversity between CCHD and HC groups (Wilcoxon rank sum test). **B** PCoA of the Bray–Curtis distances based on bacterial composition at the species level revealed significant difference between CCHD and HC groups (PERMANOVA). Bacterial taxa that significantly correlated with the PC-axes with Spearman’s correlation coefficient >  + 0.35 or <  − 0.35 (with a maximum of the top six for each quadrant) are graphed as contributors that drive the separation. The length of the arrow represents the degree of correlation to the PC-axes. The distribution and density of samples projected onto PC-axes are displayed in violin plots and assessed individually using the Wilcoxon rank sum test. **C** Fecal samples of the overall study cohort (*n* = 95) are clustered into enterotype 1 and enterotype 2 by using PCoA of the Jensen–Shannon distances at the genus level. **D** Box plots showing the relative abundance of the top genera in each enterotype (Wilcoxon rank sum test). **E** Network illustrating the correlations between enterotype enriched species. Only the top ten most abundant species enriched in each enterotype are displayed, with orange line indicating positive correlation and gray line indicating negative correlation (Spearman, *P* < 0.05, *r* > 0.6). Pie charts showing the distribution of neonates with CCHD and HCs in respective enterotypes, with the adjacent box plots showing the relative abundance of *Bifidobacterium* and *Enterococcus* in both the groups. **F** Relative abundances of 58 bacterial species responsible for discriminating the CCHD and HC groups. The genus taxonomy of each species is shown on the right.“#” indicates bacterial species enriched in HC group; whereas “†” indicates species enriched in CCHD group. **G** Co-occurrence network deduced from bacterial taxa differentially enriched in the CCHD and HC groups. Red edges indicate positive correlations and blue edges indicate negative correlations (Spearman, *P* < 0.05, *r* > 0.6). *P < 0.05, **P < 0.01, ***P < 0.001. PCoA, principal coordinates analysis; CCHD, critical congenital heart disease; HC, healthy control

We first investigated the gut bacterial composition and diversity between the two groups (CCHD and HD groups) and observed a significant increase in α-diversity in the CCHD group (Fig. 1A). In addition, principal coordinates analysis (PCoA) of bacterial composition also suggested a significant separation between the two groups, which was largely driven by a subset of bacterial species, including *Bifidobacterium ramosum*, *Bifidobacterium biavatii*, *Lactobacillus delbrueckii*, *Bacteroides caccae*, *Enterococcus asini*, *Pseudomonas oleovorans*, and *Lachnoclostridium sp.An14* (Fig. 1B). To further delineate the differences in bacterial configurations between groups, we performed enterotype analysis using unsupervised clustering at the genus level. Intriguingly, two clusters driven by a relatively high abundance of the genera *Enterococcus* (enterotype 1) and *Bifidobacterium* (enterotype 2) were identified (Fig. 1C–E). The *Bifidobacterium* enterotype was predominant in the HC group and displayed a convergent microbial community enriched with multiple *Bifidobacterium* species, whereas the CCHD group was dominated with the *Enterococcus* enterotype and showed a relatively discrete microbial community enriched with *Enterococcus*, *Klebsiella*, and *Streptococcus*. Next, we identified 58 discriminative bacterial species between the CCHD and HC groups (Fig. 1F). Compared to that in HCs, CCHD patients were characterized by 34 enriched species mainly belonging to the genera *Enterococcus* (6 species), *Enterobacter* (5 species), and *Clostridium* (four species), and by 24 depleted species mainly belonging to the genera *Bifidobacterium* (9 species), *Lactobacillus* (4 species), and *Veillonella* (4 species). Then, a species co-abundance network was constructed to provide an overview of the interplay among these discriminative bacterial species. As shown in Fig. 1G, bacterial species belonging to the same genus clade were closely correlated with each other. For instance, several *Bifidobacterium* species enriched in HC were closely correlated to generate a covarying cluster. Notably, we observed that species belonging to the genera *Bifidobacterium*, *Lactobacillus*, and *Veillonella* were positively correlated and constituted a symbiotic bacterial community in HC, whereas the species corresponding to the genera *Enterococcus*, *Enterobacter*, and *Clostridium* were discretely enriched in the CCHD group.

### Temperate core virome is implicated in early life bacterial perturbations in CCHD

The gut virome is an essential component of the human gut microbiome; however, the ecological interaction between gut viral and bacterial communities remains poorly understood. Here, we characterized the gut virome in both CCHD and HC groups, and investigated its interaction with gut bacteria. We first explored the richness of the gut virome and found a relative increase in α-diversity in the CCHD group (Additional file 1: Figure S2A). As expected, the PCoA of viral composition at the family level also revealed a distinct separation between the two groups, mainly driven by *Siphoviridae*, *Myoviridae*, and *Herelleviridae* (Additional file 1: Figure S2B). After filtering out the low-abundance taxa, 37 viral species responsible for discriminating the two groups were identified, of which 25 species were enriched in CCHD relative to that in HC (Additional file 1: Figure S2C). Notably, *Enterococcus* and *Escherichia* phages are the predominant bacteriophages among CCHD-enriched viruses, which is corresponding to the overabundance of *Enterococcus* and *Enterobacter* in CCHD group. To initially assess the ecological interaction, we compared bacterial richness with viral richness and found a strong positive correlation in the CCHD group (Additional file 1: Figure S2D). Additionally, positive correlations in community richness between *Enterococcus* phages and their bacterial host (i.e., genus *Enterococcus*) were also confirmed in both groups, with the relative abundance consistently higher in CCHD (Additional file 1: Figure S2EF).

**Fig. 2:**
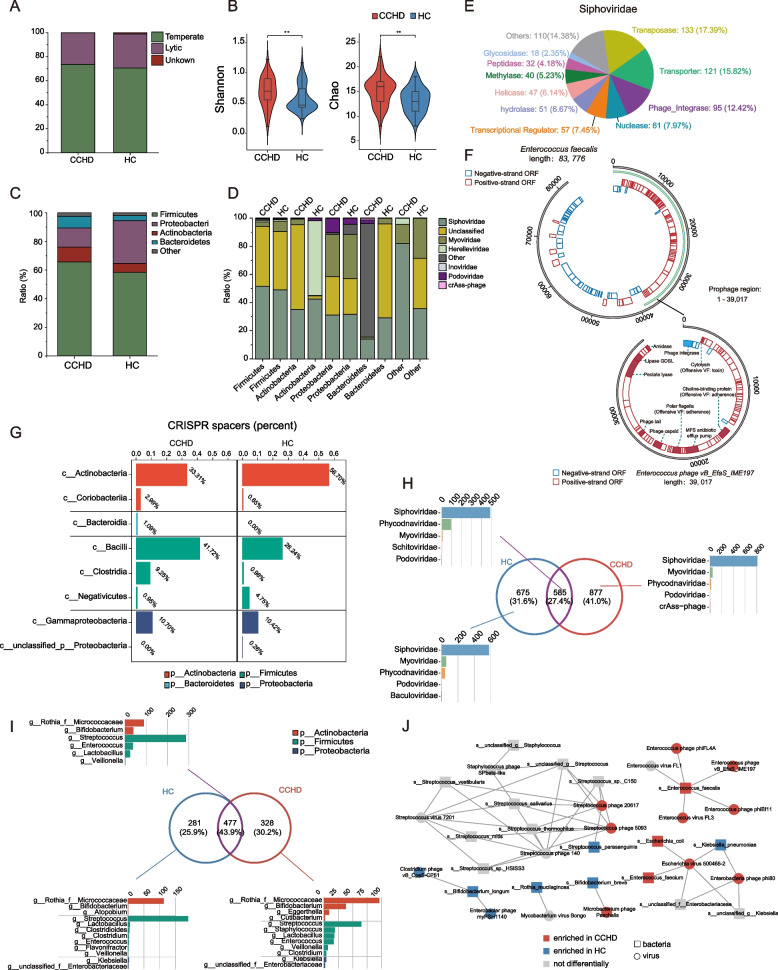
Prophage- and CRISPR-based bacteria-phage association analyses. **A** Stacked barplots showing the distribution of temperate virus and lytic virus in gut virome in both the groups. **B** The comparison of α-diversity of temperate virome between CCHD and HC groups, as assessed by using the Shannon and Chao indexes at the family level (Wilcoxon rank sum test). **C** Distribution of host-related bacterial classifications for the prophages detected in the CCHD and HC groups. **D** Viral taxonomic distribution of prophages detected from each bacterial phylum in the CCHD and HC groups. **E** Distribution of viral gene categories of *Siphoviridae* detected from *Enterococcus* contigs in CCHD group (annotated by Pfam database). **F** An example of an *Enterococcus* contig, in which the partial sequence of a viral contig was found as a prophage. **G** The distribution of detected CRISPR spacers associated with bacterial taxa at classes level in the CCHD and HC groups. **H** Venn plot showing the common and specific CRISPR spacers in the bacteriomes between CCHD and HC groups, with bar plots showing the number of viral families targeted by CRISPR spacers. **I** Venn plot showing the CRISPR spacers targeting *Siphoviridae* in the CCHD and HC groups, with bar plots showing the number of CRISPR spacers in the bacterial genera. **J** Network plot depicting the infectious relationships between bacteriophages and host bacteria, inferred from CRISPR-based bacteria–phage association analysis. Nodes are colored according to differential abundance results. The edge thickness indicates range of *P* values (*P* < 0.05). **P < 0.01. CCHD, critical congenital heart disease; HC, healthy control; CRISPR, clustered regularly interspaced short palindromic repeats

To generate an integrated view of the cross-kingdom interaction between gut virome and bacterial community during the earliest stage of life, we further investigated the replication mode of the predicted phages and conducted prophage- and clustered regularly interspaced short palindromic repeat (CRISPR)-based bacteria–phage association analyses. Notably, temperate phages were the predominant viruses in both the CCHD and HC groups, accounting for more than 70% of the viral sequences (Fig. 2A). Additionally, the α-diversity of temperate phages was higher in the CCHD group compared to HC group (Fig. 2B). Next, we analyzed prophage-based host bacteria–phage associations in samples from both the groups. In total, 935 and 694 prophages were successfully identified in the CCHD and HC groups, respectively (Fig. 2C), corresponding to a higher viral richness in CCHD (Fig. 2B). By classifying the detected prophages according to their host bacteria, we observed a similar taxonomic distribution pattern at the phylum level between the two groups (Fig. 2C). Among the four representative intestinal bacterial phyla—Firmicutes, Actinobacteria, Proteobacteria, and Bacteroidetes—the number of identified prophages was highest in Firmicutes, accounting for more than 58% of all prophages in both groups. Nevertheless, compared to those in HCs, the proportions of prophages integrated into the genomes of Proteobacteria and Bacteroidetes were significantly elevated in CCHD, whereas the proportion of Actinobacteria with prophages declined (*P* < 0.001, chi-square test, Fig. 2C). We then examined the viral taxonomy of the detected prophages based on their bacterial phyla (Fig. 2D). Generally, phages classified as *Siphoviridae* show major interactions with bacteria. For instance, 51.47% of the Firmicutes-derived prophage sequences in the CCHD group were identified as belonging to *Siphoviridae* (Fig. 2D). Notably, *Siphoviridae* has generally been identified as a temperate phage with an inherent ability to mediate the transfer of genes between bacteria and co-evolve with its host [38]. To determine whether there is a host bacteria–temperate phages co-evolution relationship mediating the overgrowth of *Enterococcus* in neonates with CCHD, we extracted *Enterococcus*-derived prophage sequences classified as *Siphoviridae* in the CCHD group and annotated the gene function (mainly the open reading frames, ORFs) using the Pfam database. Intriguingly, 56% of the annotated ORFs were classified as transposase, phage integrase, and enzymatic genes associated with transcriptional regulation and catabolism (including transcriptional regulators, methylases, hydrolases, peptidases, and glycosidases; Fig. 2E), indicating that temperate phages targeting *Enterococcus* can extensively affect the genetic makeup and metabolic traits of their bacterial hosts. Furthermore, the same prophage sequences were aligned to virulence factor database (VFDB) and comprehensive antibiotic resistance database (CARD), with the purpose to investigate whether there is a complex genetic repertoire of virulence factors and antibiotic resistance genes (ARGs) derived from temperate phages affecting the bacterial hosts’ behavior and fitness. Notably, 47.56% of the predicted ORFs were classified as virulence factor genes, and the functional genes encoding offensive virulence factors associated with adherence, toxin, and secretion system were the most frequent category (Additional file 1: Figure S3A). In addition, we identified an extensive repertoire of ARGs that may confer resistance to up to 29 types of antibiotics (Additional file 1: Figure S3B, C). The most diverse ARG type was identified as antibiotic efflux pump genes, which account for 58.43% of all annotated ARGs and demonstrate resistance to multiple antibiotics including macrolide, fluoroquinolone, tetracycline, and aminoglycoside. Besides, the dominant ARG types against distinct drug classes included macrolide (relative abundance 15.40%), tetracycline (11.59%), fluoroquinolone (10.26%), peptide antibiotic (7.37%), and penam resistance genes (5.30%). Lastly, we selected a host bacterium–prophage pair to decipher the cross-kingdom relationship (Fig. 2F). The viral sequence classified as *Enterococcus* phage vB_EfaS_IME197 (39,017-nt-long) was identified as a prophage sequence (99.6% identity) in a bacterial sequence classified as *Enterococcus faecalis* (83,776-nt-long). In addition to the phage structural protein genes, the presence of functional genes encoding virulence factors associated with adherence and toxin, enzymes associated with catabolism, and antibiotic efflux pump was confirmed in the prophage sequence.

CRISPR spacers are DNA loci that lie in the bacterial genome and act as a defensive system against phages; therefore, they can be used as a fingerprint to investigate infectious associations between gut bacteria and phages [28, 29]. Initially, we screened CRISPR spacers on the bacterial sequence using CRISPRDetect [36]. Overall, 2747 and 3072 CRISPR spacers were detected in the CCHD and HC groups, respectively, with most spacers derived from Bacilli, Actinobacteria, and Gamma-proteobacteria (Fig. 2G). Notably, compared to that in HCs, the proportion of spacers detected in Bacilli increased significantly in the CCHD group, whereas the proportion of spacers derived from Actinobacteria decreased (*P* < 0.001, chi-square test), suggesting a shift in the infectious relationship between the two groups (Fig. 2G). Next, we examined the distribution of CRISPR spacers in both the groups and the number of viral families targeted by the CRISPR spacers (Fig. 2H). Generally, almost all spacers were aligned to viral sequences classified as *Siphoviridae*. Therefore, we determined which bacterial phyla carry CRISPR spacers specific for *Siphoviridae*. CRISPR spacers against *Siphoviridae* were frequently detected for the genera *Streptococcus* and *Rothia* in both the groups (Fig. 2I), implying that *Siphoviridae* might preferentially infect bacteria belonging to Firmicutes and Actinobacteria. To systematically elucidate the infectious relationships, we constructed a network model of phage–host pairs by integrating the associations inferred from CRISPR spacers (Fig. 2J). Multiple scenarios of infectious relationships were observed, wherein certain bacterial species could be infected by different phages (or vice versa). Notably, positive relationships were observed between *Enterococcus faecalis* and several *Enterococcus* phages enriched in CCHD, further implying a co-evolutionary relationship between *Enterococcus* phages and their bacterial hosts.

Collectively, these findings suggest that a temperate core virome represented by *Siphoviridae* can act as an intrinsic force mediating the infectious kinetics and co-evolutionary relationship between gut bacteria and phages, and further implicates early life bacterial perturbations in CCHD by modifying microbial adaptation with a complex repertoire of functional genes.

### Systemic interactions between gut microbiome and fecal metabolome

Fecal metabolomics analysis can provide a functional readout of the gut microbiome. Here metabolomic profiling of paired fecal samples was conducted to characterize the metabolic signatures of both the groups. A total of 748 metabolites were identified by liquid chromatograph-mass spectrometry (LC–MS)–based untargeted metabolomics analysis. PCoA of metabolite distribution demonstrated that the overall metabolic signatures between the two groups were significantly different (Additional file 1: Figure S4A). By leveraging the orthogonal partial least-squares discriminant analysis (OPLS-DA), 120 discriminative metabolites between CCHD and HC groups were identified (Additional file 1: Figure S4B). Specifically, the CCHD group displayed enrichment of 36 metabolites and depletion of 84 metabolites compared to those in the HC group. Moreover, pathway-based differential abundance analysis highlighted that the metabolic pathways of thiamine metabolism, linoleic acid (LA) metabolism, biosynthesis of unsaturated fatty acids, galactose metabolism, phenylalanine metabolism, and tyrosine metabolism were downregulated in the CCHD group, whereas the metabolic pathways of arachidonic acid (AA) metabolism and primary bile acid biosynthesis were upregulated (Additional file 1: Figure S4C).

To explore the potential relevance between bacterial composition and metabolomic phenotypes, we calculated Spearman’s correlation matrices and constructed a co-occurrence network of differential bacterial species and metabolites (Additional file 1: Figure S5). To visualize strong associations between bacteria and metabolites, only results showing Spearman’s correlation coefficients >  + 0.6 or <  − 0.6 with a significance (i.e., *P* < 0.05) were plotted. Consistent with our initial findings, the differential bacterial species mainly generated four covarying clusters corresponding to their genus annotations (*Bifidobacterium*, *Lactobacillus*, *Enterococcus*, and *Enterobacter*), and the differential metabolites were clustered according to the metabolic pathways they were involved in. Interestingly, most bacterium–metabolite associations converged to the *Bifidobacterium*, *Lactobacillus*, and *Enterococcus* clusters. Strong positive correlations were observed between the genus *Bifidobacterium* and fecal metabolites belonging to amino acid and carbohydrate metabolism through some node species (*Bifidobacterium longum CAG:69*, *Bifidobacterium bifidum*, and *Bifidobacterium catenulatum*) and metabolites (l-arginine, N-acetyl-l-glutamate, l-fucose, and rhamnose). Notably, fecal levels of aromatic lactic acids (including hydroxyphenyllactic acid and indolelactic acid [IAA]), newly recognized probiotic-associated metabolites derived from phenylalanine and tryptophan metabolism, were positively correlated with the abundance of *Lactobacillus paragasseri* and most *Bifidobacterium* species, while consistently displaying negative correlations with *Enterococcus gallinarum*.

### Integrated network analysis of microbial abundance, functionality, and genomic structural variations with host metabolism

In addition to microbial abundance, the investigation of microbial functionality and genetic variations can provide an extra layer of information that facilitates mechanistic insights into the role of the gut microbiome in host metabolism. To this end, we first functionally profiled gene families in all metagenomes and identified 6103 Kyoto Encyclopedia of Genes and Genomes (KEGG) orthologs (KOs). Based on the PCoA of KEGG pathways, the overall microbial functionality between the two groups was significantly different (Additional file 1: Figure S6A). In addition, 840 differential KOs were identified, most of which were involved in metabolic pathways, especially amino acid metabolism, carbohydrate metabolism, and the metabolism of cofactors and vitamins (Additional file 1: Figure S6B). Linear discriminant analysis (LDA) showed that 33 metabolic pathways were differentially depleted in the CCHD group, most of which belonged to amino acid metabolism (aromatic amino acids (AAAs), branched chain amino acids, lysine, and arginine biosynthesis) and vitamin metabolism (riboflavin, vitamin B6, and pantothenate metabolism), whereas the other 29 metabolic pathways enriched in the CCHD group mainly belonged to carbohydrate and lipid metabolism, such as glycerolipid metabolism and lipopolysaccharide biosynthesis (Additional file 1: Figure S6C).

Next, we systematically detected microbial structural variations (SVs), which are highly variable segments of bacterial genomes deleted from certain species (deletion SVs, dSVs) or present in a variable number of copies (variable SVs, vSVs) in others. We identified 428 SVs from 15 bacterial species present in at least 5 samples in both the CCHD and HC groups (with an average size of 14 kbp per SV), including 157 vSVs and 271 dSVs, at a rate of 1–81 SVs per species (Additional file 1: Figure S7A, B). We further assessed the SV variability among all samples. Interestingly, we found that the genetic variability of SVs differed substantially across species, with *Escherichia coli* and *Staphylococcus hominis SK119* displaying the greatest inter-group genetic variability, whereas *Bifidobacterium animalis*, *Bifidobacterium bifidum PRL2010*, and *Bifidobacterium longum* showed relatively low inter-group genetic variability (Additional file 1: Figure S7C, D).

**Fig. 3:**
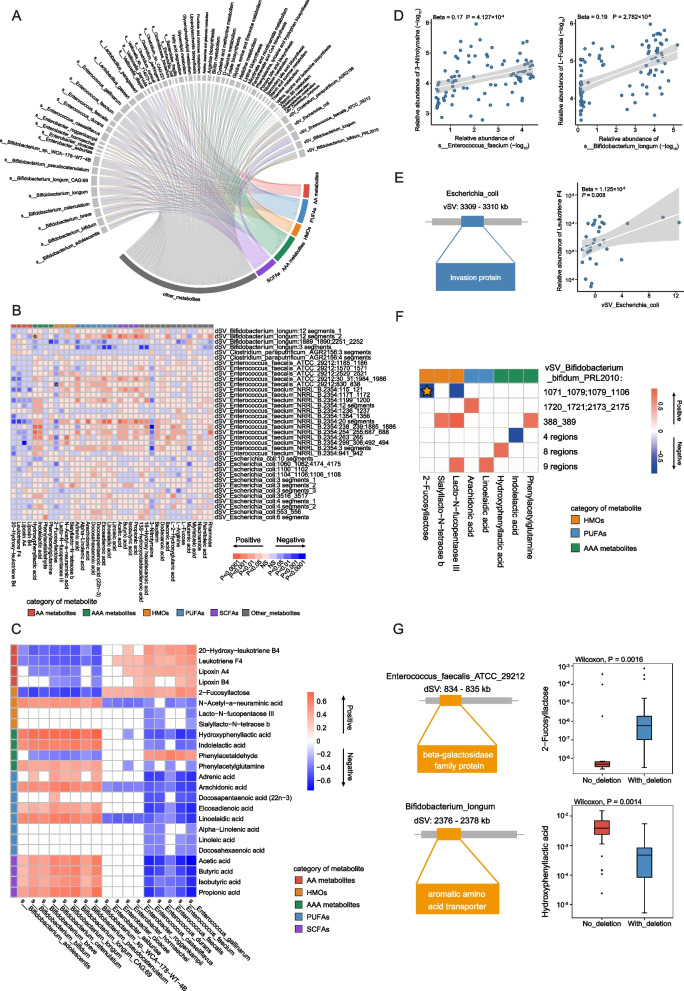
Integrated association of the differential gut microbial features with fecal metabolites, CCHD versus HC. **A**, **B** Overview of the significant associations between multilevel microbial features and fecal metabolites. The significant associations between continuous microbial variables (microbial abundance, metabolic pathways, and vSVs) and fecal metabolites are determined by Spearman’s rank correlation analysis and shown in Circos plot **A**; whereas the significant associations between binary microbial variables (dSVs) and fecal metabolites are determined by Mann–Whitney *U* test and shown in heatmap **B**. The associated microbial features and metabolites are identified in the comparison of CCHD and HC. Other metabolites include lipids, amino acids, nucleotides, and carbohydrates. **C** Heatmap of multiple associations between microbial abundance (*Bifidobacterium*, *Enterobacter*, and *Enterococcus*) and fecal metabolites (Spearman’s rank correlation analysis). Only significant associations are plotted. **D** Scatter plots showing positive associations between *Enterococcus faecium* and 3-nitrotyrosine and *Bifidobacterium longum* and l-fucose. **E** Scatter plot showing positive association between a 2-kbp vSV in the *Escherichia coli* genome and leukotriene F4. **F** Heatmap of multiple associations between vSVs in *Bifidobacterium bifidum PRL2010* and fecal metabolites (Spearman’s rank correlation analysis). Only significant associations are plotted. **G** Boxplots (center, median; box, IQR; whiskers, IQR × 1.5; dots, outliers) showing the relative abundance of 2-fucosyllactose in individuals harboring an 8-kbp dSV in the *Enterococcus faecalis ATCC29212* genome (blue, *n* = 18) and individuals with no deletion (red, *n* = 18), and the relative abundance of hydroxyphenyllactic acid in individuals harboring a 21-kbp dSV in the *Bifidobacterium longum* genome (blue, *n* = 8) and individuals with no deletion (red, *n* = 40). Significance is determined by Mann–Whitney *U* test. CCHD, critical congenital heart disease; HC, healthy control; AA, arachidonic acid; HMOs, human milk oligosaccharides; AAA, aromatic amino acid; PUFAs, polyunsaturated fatty acids; SCFAs, short chain fatty acids; IQR, interquartile range; dSV, deletion structural variation; vSV, variable structural variation

Then, we explored the associations between multilevel microbial features (bacterial abundance, functionality, and SVs) and fecal metabolites. These investigation identified 1776 significant associations between 142 microbial features and 56 metabolites, including 1161 associations with species and pathway abundance and 615 associations with microbial SVs (Fig. 3A, B). To simplify the intricate correlation network of bacteria and metabolites, we deconstructed and clustered the associations according to metabolite categories and mainly focused on associations with AA metabolites [39, 40], polyunsaturated fatty acids (PUFAs) [41, 42], human milk oligosaccharides (HMOs) [43, 44], AAA metabolites [45], and short-chain fatty acids (SCFAs) [46], all of which are well-known gut microbiome-related metabolites implicated in host health.

Notably, > 60% of the significant associations between metabolites and bacterial abundance were attributed to the genera *Bifidobacterium* and *Enterococcus*. By plotting the associations between the abundance of *Bifidobacterium*, *Enterococcus*, and the metabolites of interest, we observed a “mutually exclusive” characteristic of associations with *Bifidobacterium* and *Enterococcus* (Fig. 3C). Specifically, most *Enterococcus* species were consistently positively correlated with AA metabolite levels (20-hydroxy-leukotriene B4, leukotriene F4, lipoxin A4, and lipoxin B4), whereas inverse associations were concomitantly found between *Enterococcus* and PUFA levels (eicosadienoic acid, docosahexaenoic acid, and LA; Fig. 3C). Notably, all these AA metabolites are active compounds involved in the inflammatory cascades [39], whereas the ω-3 and ω-6 PUFAs are capable of alleviating inflammation and oxidative stress [47]. Intriguingly, a significant positive correlation was also identified between the abundance of *Enterococcus faecium* and 3-nitrotyrosine (Fig. 3D), which is an oxidative product of tyrosine and a biomarker of inflammation and oxidative stress [48].

We then looked at the correlations between microbial SVs and fecal metabolites (Fig. 3A, B). Notably, 29.9% (184 out of 615) of the associations between microbial SVs and metabolites were related to the dSVs of *Enterococcus faecium NRRL B.2354*, a genetically unstable species identified in our initial findings (Additional file 1: Figure S6C). Although many SVs harbor genes with unknown functions, we observed several intriguing functions in metabolite-associated SVs. For instance, a 2-kbp vSV in *Escherichia coli* that contains genes encoding invasion proteins was positively correlated with the abundance of leukotriene F4 (Fig. 3E), suggesting that genetic variation in certain functional genes may be implicated in microbial adaptation and the subsequent activation of the host inflammatory response.

Next, we focused on the correlations between differential microbial features and HMOs. Impressively, negative correlations were observed between the abundance of 2-fucosyllactose (2′-FL) and most *Bifidobacterium* species, as well as a vSV of two segments in *Bifidobacterium bifidum PRL2010* (Fig. 3F). Moreover, the abundance of *Bifidobacterium longum* was strongly positively correlated with l-fucose (Fig. 3D), which is a microbial catabolite of 2′-FL with immunoregulatory activity. Fucosyllactose-utilization genes are suggested to imprint the early life development of the gut microbiome in infants [49]. This prompted us to perform an in-depth analysis of HMO-utilization genes in fecal metagenomes. Intriguingly, a total of 37 HMO-utilization genes were identified and assigned to five clusters, which were all over-represented in the HC group relative to that in the CCHD group (Additional file 1: Figure S8A), and most of them were inversely correlated with 2′-FL abundance (Additional file 1: Figure S8B). By contrast, the overgrowth of *Enterococcus*, the depletion of HMO-utilization genes in CCHD, and an 8-kbp dSV that contains genes encoding beta-galactosidase family proteins in *Enterococcus faecalis ATCC29212* were positively correlated with 2′-FL abundance (Fig. 3C, G, Additional file 1: Figure S8), indicating the incapability of HMO-utilization.

Another interesting category of metabolites is the aromatic lactic acids, and we observed that the abundance of most *Bifidobacterium* species was positively correlated with these metabolites (Fig. 3C). Furthermore, the metabolic pathway of phenylalanine, tyrosine, and tryptophan biosynthesis also demonstrated a remarkable positive correlation with IAA (Additional file 1: Figure S9). As a noteworthy example, a 21-kbp dSV in *Bifidobacterium longum*, which contains genes coding for the enzyme AAA transporter, was positively associated with hydroxyphenyllactic acid abundance (the median level was higher for retention, adjusted *P* < 0.01, *n* = 48, 40 retaining; Fig. 3G).

Collectively, these findings indicate that the aberrant gut microbial composition in neonates with CCHD, characterized by the overgrowth of *Enterococcus* and depletion of *Bifidobacterium*, together with the alterations in microbial functionality and genetic makeup, considerably impact early life immune development and metabolism. Moreover, microbial metabolites are important agents involved in host–microbe interactions. An in-depth analysis of microbial genetics provides mechanistic insights into the metabolomic perturbations.

### Overgrowth of Enterococcus was related to inflammatory response and poor prognosis

To further investigate the role of the gut microbiome in clinical outcomes of neonates with CCHD, we first separated our patient cohort into two subgroups based on surgical prognosis and conducted a comparative analysis. Specifically, composite adverse events were used to define the poor prognosis (Additional file 1: Table S2). Nineteen patients who had one or more adverse event/s were classified as having a poor prognosis. The perioperative clinical metadata are summarized in Additional file 1: Table S3. In brief, no significant differences were observed in the demographic data and preoperative and intraoperative variables, including median age at operation, blood oxygen saturation (SpO_2_), cardiac surgery complexity (RACHS-1 and ABC categories), cardiopulmonary bypass (CPB) time, and intraoperative blood loss.

**Fig. 4:**
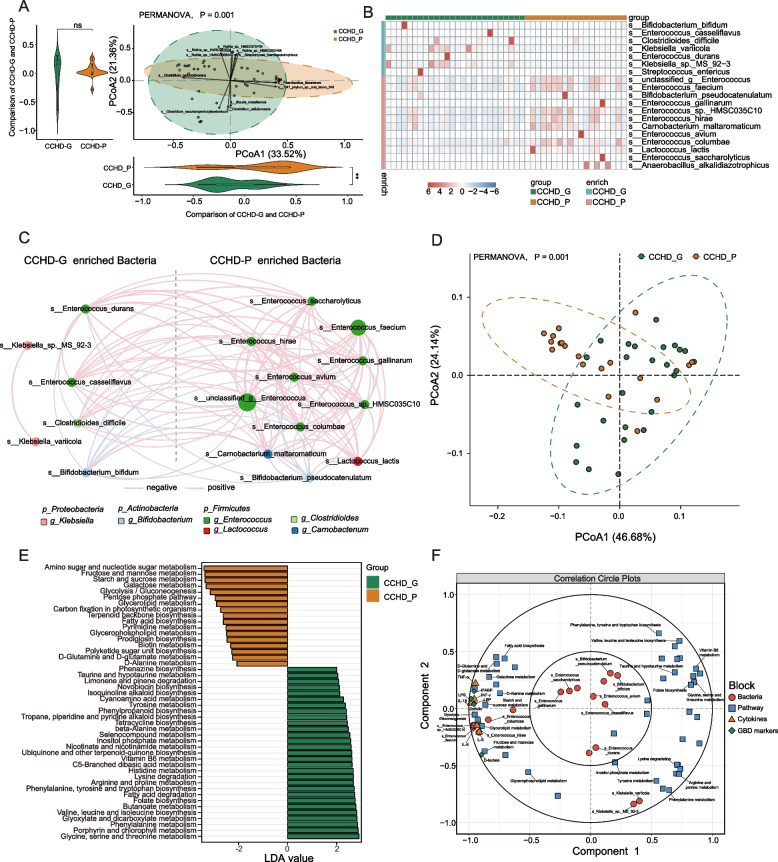
Aberrant gut microbiome in CCHD patients is associated with gut barrier impairment and systemic inflammation. **A** PCoA of the Bray–Curtis distances based on bacterial composition at the species level revealed significant differences between the CCHD-P and CCHD-G subgroups (PERMANOVA). Bacterial taxa that significantly correlated with the PC-axes with Spearman’s correlation coefficient >  + 0.4 or <  − 0.4 (with a maximum of the top six for each quadrant) are graphed as contributors that drive the separation, and the length of the arrow represents the degree of correlation to the PC-axes. The distribution and density of samples projected onto PC-axes are displayed in violin plots and assessed individually using the Wilcoxon rank sum test. **B** Heatmap of discriminative bacterial species between CCHD-P and CCHD-G subgroups. **C** Co-occurrence network deduced from bacterial taxa differentially enriched in the CCHD-P and CCHD-G subgroups. The size of each node is proportional to the mean relative abundance. Red edges indicate positive correlation and blue edges indicate negative correlation (Spearman, *P* < 0.05, *r* > 0.4). **D** PCoA based on the relative abundance of the KEGG orthology groups revealed significant differences in the microbial functionality between CCHD-P and CCHD-G subgroups (PERMANOVA). Dashed lined ellipses indicate 95% confidence interval (CI) of datapoints. **E** LDA showing discriminative KEGG metabolic pathways between CCHD-P and CCHD-G subgroups. **F** Correlation circle plot showing associations between multiple categories of variables (including the relative abundance of bacterial species, KEGG metabolic pathways, and biomarkers of intestinal permeability and systemic inflammation), as measured via canonical correlations. Neighboring of variables indicates correlations between variables. **P < 0.01; NS, not significant; CCHD-G, CCHD patients with good prognosis; CCHD-P, CCHD patients with poor prognosis; PCoA, principal coordinates analysis; LDA, linear discriminant analysis; GBD, gut barrier dysfunction

Next, we examined the gut microbial composition between HCs and patients with good and poor surgical prognosis (termed CCHD-G and CCHD-P respectively). Intriguingly, pairwise comparative analyses revealed significant differences in gut microbial configurations between the three groups (Fig. 4A, Figure S10A–D), with LDA indicating that *Enterococcus* species were enriched in both CCHD-P and CCHD-G when compared to HC (Additional file 1: Figure S10E, F). More notably, a total of 19 discriminative bacterial species were identified between CCHD-P and CCHD-G, with 14 species enriched in CCHD-P mainly belonging to genus *Enterococcus* (eight species; Fig. 4B), implying that the overgrowth of *Enterococcus* is a crucial microbial feature that drives the separation between CCHD-P and CCHD-G. To comprehensively characterize the microbial features between the two subgroups, we constructed a co-abundance network based on the differential taxa and observed a synergistic microbial consortium of *Enterococcus* harbored in CCHD-P (Fig. 4C). Accordingly, the overall microbial functionality between the two subgroups was significantly different (Fig. 4D). By leveraging LDA, 29 metabolic pathways were found to be downregulated in CCHD-P, mainly associated with amino acid metabolism (eight pathways) and metabolism of cofactors and vitamins (five pathways), whereas the other 17 metabolic pathways upregulated in CCHD-P mainly belonged to carbohydrate and lipid metabolism (six pathways, Fig. 4E).

Interestingly, these findings were in accord with the initial evidence we observed from the comparative analysis between CCHD patients and HCs, which further prompted us to hypothesize that gut microbiota dysbiosis in neonates with CCHD, characterized by the overgrowth of *Enterococcus*, is implicated in worsening surgical outcomes by mediating inflammatory responses and microbial metabolites. To verify this hypothesis, we first examined the inflammatory status of the patients. Blood samples collected on admission were used to quantify the serum levels of inflammatory cytokines (including interleukin-1β [IL-1β], interleukin-6 [IL-6], interleukin-8 [IL-8], tumor necrosis factor-α [TNF-α], and interferon-γ [INF-γ]) and biomarkers of intestinal permeability (including zonulin, D-lactate, intestinal fatty acid binding protein [iFABP], lipopolysaccharide [LPS], and lipopolysaccharide binding protein [LBP]) [13, 50, 51]. Notably, although at low titers, the serum levels of all these biomarkers were significantly increased in CCHD-P relative to those in CCHD-G (Additional file 1: Figure S11, S12), indicating that subclinical systemic inflammation and gut barrier impairment existed prior to cardiac surgery in neonates with poor prognosis. To investigate an aberrant gut microbiota-disrupted gut barrier–systemic inflammation axis, we employed an integrated correlation analysis of differential microbial features and serum biomarkers of inflammation and intestinal permeability. Notably, strong positive associations were observed between *Enterococcus* abundance and the serum levels of multiple proinflammatory cytokines and biomarkers of intestinal permeability (Fig. 4F). Furthermore, univariable and multivariable logistic regression analyses were performed to investigate whether there is predictive value of *Enterococcus* species in prognostic stratification for neonates with CCHD (independent of traditional clinical risk factors, e.g., SpO_2_, cardiac surgery complexity indexes, and CPB time, Additional file 1: Table S4). Intriguingly, a predictive model comprising of *Enterococcus faecium* abundance, CPB time, and intraoperative infusion volume was constructed and yielded an area under the curve (AUC) of 0.86 (95% CI 0.74–0.97) in the study cohort (Additional file 1: Figure S13), indicating that *Enterococcus faecium* abundance could be an independent predictor of surgical prognosis for neonates with CCHD.

### Microbial metabolites linked Enterococcus to the immune–inflammatory imbalance

By profiling the fecal metabolites of both subgroups, we identified significant differences in the overall metabolic signatures between the two subgroups (Additional file 1: Figure S14A, B). Accordingly, pathway-based differential abundance analysis revealed that most metabolic pathways associated with amino acids, vitamins, and unsaturated fatty acids were downregulated in the CCHD-P group (Additional file 1: Figure S14C). To initially investigate the pivotal bacterial species that were highly associated with metabolomic alterations, we constructed a co-abundance network of differential bacteria and metabolites. Notably, the interplay between bacteria and metabolites was largely attributed to the negative associations between CCHD-P-enriched *Enterococcus* species and metabolites involved in lipid and vitamin metabolism (Additional file 1: Figure S15), indicating that antagonistic relationships between *Enterococcus* and lipid and vitamin metabolism could be central to the metabolomic perturbations in neonates with poor prognosis.

**Fig. 5:**
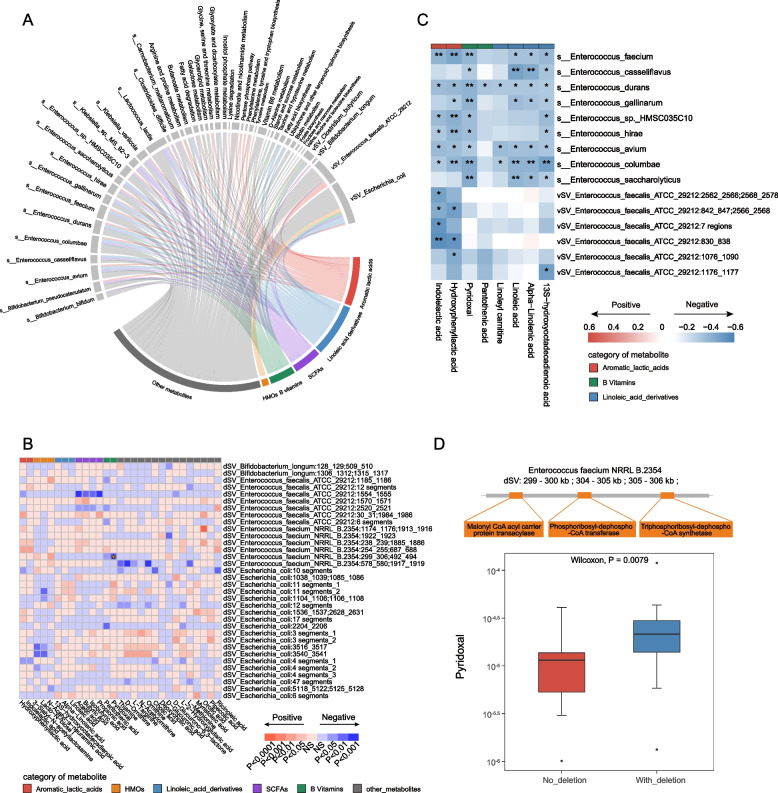
Integrated association of the differential gut microbial features with fecal metabolites, CCHD-P versus CCHD-G. **A**, **B** Overview of the significant associations between multilevel microbial features and fecal metabolites. The significant associations between continuous microbial variables (microbial abundance, metabolic pathways, and vSVs) and fecal metabolites are determined by Spearman’s rank correlation analysis and shown in Circos plot **A**; whereas the significant associations between binary microbial variables (dSVs) and fecal metabolites are determined by Mann–Whitney *U* test and shown in heatmap **B**. The associated microbial features and metabolites are identified in the comparison of CCHD-P and CCHD-G. Other metabolites include lipids, amino acids, nucleotides, and carbohydrates. **C** Heatmap of significant correlations between fecal metabolites (including LA derivatives, B vitamins, and aromatic lactic acids) with *Enterococcus* abundance and vSVs in *Enterococcus faecalis ATCC29212* (Spearman’s rank correlation analysis). Asterisks indicate statistical significance. **D** Boxplots (center, median; box, IQR; whiskers, IQR × 1.5; dots, outliers) showing the relative abundance of pyridoxal in individuals harboring a 9-kbp dSV in *Enterococcus faecium NRRL B.2354* genome (blue, *n* = 23) and individuals with no deletion (red, *n* = 13). Significance is determined by Mann–Whitney *U* test. *P < 0.05, **P < 0.01. CCHD-G, CCHD patients with good prognosis; CCHD-P, CCHD patients with poor prognosis; HMOs, human milk oligosaccharides; SCFAs, short-chain fatty acids; LA, linoleic acid; dSV, deletion structural variation; vSV, variable structural variation

To further expand our understanding of the interactions between the aberrant gut microbiome and metabolomic alterations within neonates with different surgical outcomes, we performed an integrated correlation analysis of microbial features and fecal metabolites. Similarly, we focused on the associations between aromatic lactic acids, LA derivatives, SCFAs, B vitamins, and HMOs (Fig. 5A, B). As expected, we found that *Enterococcus* abundance and five vSVs in *Enterococcus faecalis ATCC 29,212* were consistently negatively associated with aromatic lactic acid levels (Fig. 5C). Intriguingly, LA and its derivatives, including 13S-hydroxyoctadecadienoic acid (13-HODE) and α-linolenic acid, were another subset of metabolites that displayed accordant reverse correlations with *Enterococcus* abundance (Fig. 5C). Another noteworthy category of metabolites, B vitamins, especially pantothenic acid (vitamin B5) and pyridoxal (vitamin B6), were highly correlated with *Enterococcus* abundance. For instance, pyridoxal was inversely correlated with the abundance of most *Enterococcus* species and a 9-kbp dSV in *Enterococcus faecium NRRL B.2354* (the median level was lower for retention, adjusted *P* < 0.01, *n* = 36, 13 retaining; Fig. 5C, D). This SV contains genes encoding the enzymes CoA synthetase, CoA transferase, and acyl-CoA, which might be involved in vitamin B6 metabolism. Pyridoxal is a critical coenzyme involved in the synthesis of amino acids and neurotransmitters (serotonin and norepinephrine), and its depletion is increasingly linked to the inflammatory response [52].

Collectively, these findings indicate that the overgrowth of *Enterococcus* together with genetic variations is highly associated with the depletion of probiotic-associated metabolites, especially aromatic lactic acids, LA derivatives, and B vitamins, thereby implicating an active inflammatory response.

### Microbiome contributed to host inflammatory response and gut barrier impairment through metabolites

**Fig. 6:**
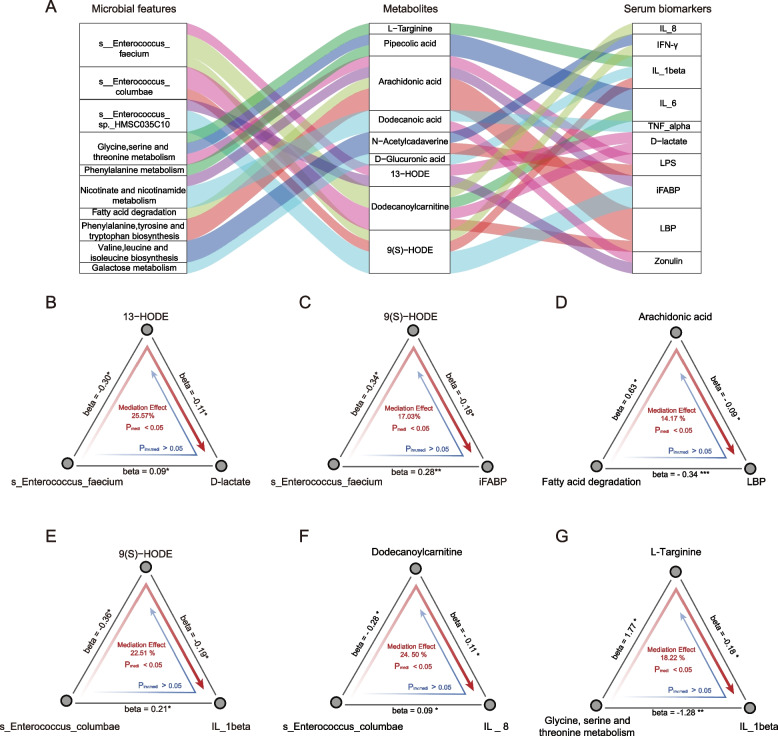
Mediation linkages among the gut microbiome, metabolites, and serum biomarkers of intestinal permeability and inflammation. **A** Sankey diagram illustrating the 23 significant mediation linkages among gut microbiome, fecal metabolites, and serum biomarkers of intestinal permeability and inflammation. Columns from left to right show microbial features (including microbial abundance and metabolic pathways), fecal metabolites, and serum biomarkers, respectively. The curved lines across the columns indicate the mediation effects and the colors correspond to different biomarkers. **B**–**G** Examples of mediation linkages among gut microbiome, metabolites, intestinal barrier dysfunction, and systemic inflammation inferred by bi-directional mediation analysis. The beta coefficient and significance are labeled at each edge, and the proportions of mediation effects are labeled at the center of the ternary diagrams. The red arrows indicate the gut microbial effects on serum biomarkers mediated by metabolites, whereas the blue arrows indicate the reverse mediation effects, that is, the microbial effects on metabolites mediated by serum biomarkers. *P*_medi_ and *P*_inv.medi_ are estimated by using the bi-directional mediation analysis. Asterisks indicate statistical significance for all panels: *P < 0.05, **P < 0.01, ***P < 0.001. 13-HODE, 13S-hydroxyoctadecadienoic acid; 9(S)-HODE, α-dimorpholic acid; IL, interleukin; IFN-γ, interferon-γ; TNF-α, tumor necrosis factor-α; LPS, lipopolysaccharide; iFABP, intestinal fatty acid binding protein; LBP, lipopolysaccharide binding protein

To further evaluate whether metabolites can mediate the microbial impact on the inflammatory response and gut barrier impairment in neonates with CCHD, we performed a bi-directional mediation analysis and revealed 23 mediation linkages (*P*_mediation_ < 0.05, *P*_inverse mediation_ > 0.05, Fig. 6A). Interestingly, most of these linkages were related to *Enterococcus faecium* (four linkages), *Enterococcus columbae* (three linkages), and the microbial functionality of glycine, serine, and threonine metabolism (three linkages). Our mediation analysis suggested that *Enterococcus faecium* might contribute to gut barrier impairment (characterized by increased serum levels of D-lactate and iFABP) by decreasing fecal levels of 13-HODE (26%, *P*_mediation_ < 0.05, Fig. 6B) and α-dimorphecolic acid (9(S)-HODE; 17%, *P*_mediation_ < 0.05, Fig. 6C), both of which are LA derivatives. Furthermore, the microbial fatty acid degradation pathway may also contribute to decreased intestinal permeability by affecting fecal AA level (14%, *P*_mediation_ < 0.05, Fig. 6D).

We also identified several metabolite mediation effects on the microbial impact on systemic inflammation. An interesting example here is *Enterococcus columbae*, a bacterium enriched in CCHD-P, which may contribute to the systemic inflammatory response by decreasing fecal levels of 9(S)-HODE (23%, *P*_mediation_ < 0.05, Fig. 6E) and dodecanoylcarnitine (25%, *P*_mediation_ < 0.05, Fig. 6F). By contrast, the microbial functionality of glycine, serine, and threonine metabolism probably contributes to alleviating the systemic inflammatory response through l-targinine (an arginine derivative; 18%, *P*_mediation_ < 0.05, Fig. 6G).

## Discussion

CCHD is a composite of complex inborn heart defects that imposes a huge burden on quality of life. In addition to multiple clinical factors being associated with CCHD prognosis, the gut microbiota remains an important but yet lesser-known aspect that likely influences the pathophysiology of CCHD. Given the critical role of the early life gut microbiome in maintaining host metabolic and immune homeostasis, we undertook in-depth analyses to elucidate the implications of the gut microbiome in neonates with CCHD.

To the best of our knowledge, this study represents the first and most in-depth analysis of the early life gut microbiome in neonates with CCHD in the Chinese population. By integrating multilevel microbial features in association with fecal metabolites and host phenotypes, we demonstrated that a disrupted gut microbiome associated with metabolomic perturbations was involved in immune imbalance and adverse clinical outcomes for CCHD patients (Additional file 1: Figure S16). Specifically, we found that a microbial consortium of probiotics, including *Bifidobacterium*, *Lactobacillus*, and *Veillonella*, was depleted in neonates with CCHD, whereas multiple opportunistic pathogens, such as *Enterococcus*, *Enterobacter*, and *Clostridium*, were enriched in CCHD patients compared to that in HCs. Depletion of *Bifidobacterium* and overgrowth of *Enterococcus* identified in CCHD were highly correlated with a panel of fecal metabolites, including HMOs, SCFAs, aromatic lactic acids, PUFAs, and B vitamins, all of which are well-recognized probiotic-associated metabolites implicated in intestinal homeostasis. Furthermore, we systematically characterized the microbial genetic variations and showed that the depletion of HMO-utilization genes and genetic SVs in *Bifidobacterium* and *Enterococcus* orchestrated the metabolomic perturbations in CCHD. By leveraging prophage- and CRISPR-based association analysis, we identified a temperate core virome represented by *Siphoviridae* in neonates with CCHD, which may be implicated in shaping gut bacterial composition by modifying microbial adaptation, potentially enabling the excessive colonization of *Enterococcus* in CCHD. Moreover, we profiled serum levels of proinflammatory cytokines and biomarkers of intestinal permeability in a patient cohort to gain mechanistic insights into the involvement of the gut microbiome in CCHD pathology. Our results indicate that the overgrowth of *Enterococcus* in neonates with CCHD is highly associated with gut barrier impairment and excessive inflammatory responses, thereby implicating in poor surgical prognosis. Finally, bi-directional mediation analysis revealed mediation linkages between gut microbiota, bacteria-derived metabolites, and host pathological phenotypes, including immune–inflammatory imbalance and gut barrier impairment.

Most notably, the disrupted host–microbiome crosstalk we identified in CCHD patients essentially converged to the depletion of *Bifidobacterium* and overgrowth of *Enterococcus*. Interestingly, a prevailing theory derived from several pioneering studies suggests that the assembly of the neonate gut microbiome follows a stepwise pattern [53, 54], wherein obligate anaerobes such as *Bifidobacterium* would gradually replace the facultative anaerobic bacteria (e.g., *Enterobacter* and *Enterococcus*) and predominate in the neonate gut within the first month of life. From this perspective, the aberrant gut microbiome in CCHD patients could be interpreted as a stunted microbial configuration deviating from the normal trajectory of early life microbial succession, resulting from the delayed or failed colonization of *Bifidobacterium*, along with the overdue colonization of *Enterococcus*. Indeed, multiple early life events, such as delivery mode, breastfeeding, and environmental exposure, could steer the establishment of the early life gut microbiome [55–57]. Given that breast milk is the primary source of *Bifidobacterium* in neonates, whereas *Enterococcus* is ubiquitously detected in wards [58], we speculate that breastfeeding intolerance and prolonged exposure to the hospital environment could be crucial determinants of the aberrant gut microbiome in neonates with CCHD; however, further research is necessary to verify this speculation.

*Bifidobacterium* is among the first microbial colonizers that predominate in the gut of breastfed term neonates [59] and has long been hypothesized to exert a protective effect in mediating intestinal homeostasis. Several studies have highlighted a link between the loss of *Bifidobacterium* in infants and enteric inflammation in early life [2, 60]. Thus, the depletion of *Bifidobacterium* in CCHD may play a critical role in immune imbalance. Consistent with the results of previous research [61], we identified strong correlations between *Bifidobacterium*, SCFAs, and 2′-FL, which is the most abundant HMO derived from breast milk. Furthermore, our analysis of HMO-utilization genes further corroborated the saccharolytic activity of *Bifidobacterium* in the fermentation of 2′-FL. Notably, HMOs are non-digestible and structurally complex carbohydrates with significant abundance in breast milk, which serve as a carbon and energy source for *Bifidobacterium*. In addition to facilitating the growth of *Bifidobacterium*, HMOs can be degraded by *Bifidobacterium* to produce SCFAs, which results in a more acidic gut luminal environment and inhibits colonization by pathogenic microorganisms. SCFAs produced by *Bifidobacterium* are known to have potent anti-inflammatory properties by promoting the polarization of Treg cells in the colon and serving as fuels for nourishing colonic epithelial cells, thereby fortifying the gut barrier [62]. Aromatic lactic acids are another subset of metabolites positively correlated with *Bifidobacterium*. These bioactive compounds derived from microbial AAA metabolism have been implicated in immune homeostasis with multiple functions, including protection against pathogenic organisms, promotion of immune development, and enhancement of the intestinal barrier function [2, 45, 63, 64]. Collectively, our findings suggested that *Bifidobacterium* could substantially affect host immune homeostasis early in life via the production of SCFAs and aromatic lactic acids, whereas the depletion of *Bifidobacterium* and HMO-utilization genes could lead to an aberrant inflammatory response in CCHD patients, which further provide mechanistic insights into the supplementation of *Bifidobacterium* and non-digestible oligosaccharides to reconstitute optimal gut microbiome and immune homeostasis. Although controversial results are reported in some studies, it has been extensively shown that the addition of non-digestible oligosaccharides to infant formula has a bifidogenic effect [65], thereby reconfiguring the gut microbiota of formula-fed infants and increasing the production of SCFAs. Therefore, supplementing *Bifidobacterium* with non-digestible oligosaccharides may be a promising therapeutic strategy for reconstituting immune homeostasis in neonates with CCHD.

Our results demonstrated that the overgrowth of *Enterococcus* could induce gut barrier impairment and an excessive inflammatory response in neonates with CCHD, thereby leading to adverse clinical outcomes. Specifically, we observed positive correlations between *Enterococcus* and AA metabolites, which are active compounds involved in inflammatory cascades. Furthermore, strong positive correlations were also identified between *Enterococcus* and serum proinflammatory cytokine levels in the CCHD group, corroborating the aberrant microbial links to the inflammatory response. Indeed, accumulating evidence has emphasized a causal link between enterococcal domination and systemic inflammation in other clinical conditions [66, 67]. It is postulated that *Enterococcus* can express metalloproteases to disrupt the intestinal barrier and translocate into the bloodstream in vulnerable hosts, thereby triggering systemic inflammation [68]. In addition, a novel study in experimental rodent models suggested that the prescription of vancomycin or ampicillin could alleviate the excessive inflammatory response induced by *Enterococcus* and improve survival [69]. However, antibiotic usage carries the risk of killing beneficial bacteria and promoting dysbiosis in the clinical setting. Therefore, the development of strategies to specifically terminate *Enterococcus* expansion is essential to alleviate the inflammatory response and improve clinical outcomes of CCHD patients.

Beyond microbial abundance, SVs in microbial genomes have recently been recognized as an extra layer of variability in the gut microbiome that is closely associated with host health [22, 70, 71]. We conducted an SV-based metagenome-wide association analysis and demonstrated that this is instrumental in providing mechanistic insights into the role of microbial functionality in host pathophysiology. For instance, we noted that the deletion of a 21-kbp segment in the genome of *Bifidobacterium longum*, which contains genes hypothesized to encode proteins that enable the transport of AAAs, was correlated with a lower fecal level of hydroxyphenyllactic acid, which is thought to be implicated in host immune homeostasis [45]. Another interesting example is that the copy number of a 2-kbp segment in the genome of *Escherichia coli*, which contains genes encoding a virulence factor, was positively correlated with the fecal leukotriene F4 level, which is an indicator of innate immune response [72]. Collectively, our data suggest mechanisms underlying the role of microbial genetic variation in host immunity.

There is limited understanding of the ecological interaction between gut viral and bacterial communities during the earliest stage of life. Consistent with the results of previous research, we showed that temperate phages are the predominant viruses within the gut virome of neonates [73]. Interestingly, positive associations in richness between gut bacteria and phages were identified at both the individual and population levels, with prophage- and CRISPR-based association analyses further indicating the co-evolutionary relationship between temperate phages and their bacterial hosts. Most notably, a temperate core virome represented by *Siphoviridae* was identified with a complex repertoire of functional genes, which may be implicated in modulating microbial adaptation and enabling the overabundance of *Enterococcus* in neonates with CCHD. Indeed, beyond the well-known predator–prey relationship which has generally been observed between lytic phages and bacteria [74], temperate phages interact with their bacterial hosts in intricate ways, as they can integrate into bacterial genomes and affect hosts’ behavior via horizontal gene transfer [38]. Moreover, recent evidence shows that temperate phages will become increasingly more abundant in ecosystems with high microbial densities, further indicating the parasitic propensity of temperate phages [75]. Although the study of host bacteria–temperate phages co-evolution is still in its infancy, accumulating evidence suggests that temperate phages can be mutualistic with their bacterial hosts as well [38]. Given their inherent ability to integrate into bacterial genomes, temperate phages can introduce a plethora of genes that provide functions of different kinds to their hosts, which can expand the bacterial hosts’ metabolic repertoire [76, 77], confer or enhance virulence [78], or eliminate competing organisms by mediating bacteriocin release [79], and thus enhance bacterial fitness. Moreover, temperate phages can facilitate biofilm formation leading to population-level benefits for their bacterial hosts [80]. Thus, our series of analyses corroborates the mutualistic relationship between temperate phages and host bacteria and demonstrates that metagenomic analysis of gut virome combined with prophage- and CRISPR-based association analysis could be useful to explore the cross-kingdom interaction between gut viruses and bacteria. However, further studies with deeper analyses and experiments are needed to confirm our findings. Besides, in view of the tightly intertwined co-evolutionary dynamics of phages and bacteria, future research with more metagenomic information on intestinal phages and bacteria will open the possibility of developing phagotherapy to specifically manipulate gut microbiome dysbiosis in neonates with CCHD.

In summary, our study provides the first evidence to demonstrate that gut microbiome dysbiosis is closely correlated with metabolomic perturbations in neonates with CCHD and that an aberrant gut microbiome may cause an excessive inflammatory response, thereby leading to adverse clinical outcomes for CCHD patients. These findings highlight the importance of reconstituting optimal host–microbe interactions in CCHD to prevent adverse clinical outcomes. Given that limited therapeutic strategies are currently available to effectively prevent perioperative infection and alleviate excessive inflammatory response and antibiotic prophylaxis may further deteriorate gut microbial disturbances, our research is the first to advocate for an individualized therapeutic strategy targeting the gut microbiota to restore metabolic and immunological homeostasis and improve clinical outcomes for CCHD.

We acknowledge several limitations in our study. First, the patient cohort of this study was comprised neonates with CCHD, and whether these findings may be applicable to other types of CHD remains unknown. Future research on the entire spectrum of CHD is required to provide a comprehensive understanding of the impacts of the gut microbiota in CHD. Second, this study was observational and the associations were not proof of causation, although we did perform bi-directional mediation analysis to infer the mediation linkages. Moreover, we provided some potential biological mechanistic hypotheses by integrating multi-omics datasets, including metagenomics and metabolomics, which require further experimental validation.

## Conclusions

We demonstrate for the first time that an aberrant gut microbiome associated with metabolomic perturbations is implicated in immune imbalance and adverse clinical outcomes in neonates with CCHD. Our data support the importance of reconstituting optimal gut microbiome in maintaining host metabolic and immunological homeostasis in CCHD.

## Supplementary Information

**Additional file 1: Supplementary file 1.**

## Data Availability

The metagenomic sequencing raw data generated in this study have been deposited in Genome Sequence Archive for Human in the National Genomics Data Center (https://ngdc.cncb.ac.cn/bioproject) under BioProject accession number PRJCA010520. R code used to analyze the data and generate the manuscript figures is available within the supplementary file. All other relevant data supporting the findings of this study are available from the corresponding author upon reasonable request.

## Declarations

### Provenance and peer review

Not commissioned, externally peer reviewed.

## References

[1] Ronan V, Yeasin R, Claud EC (2021). Childhood development and the microbiome-the intestinal microbiota in maintenance of health and development of disease during childhood development. Gastroenterology.

[2] Henrick BM, Rodriguez L, Lakshmikanth T, Pou C, Henckel E, Arzoomand A (2021). Bifidobacteria-mediated immune system imprinting early in life. Cell.

[3] Planer JD, Peng Y, Kau AL, Blanton LV, Ndao IM, Tarr PI (2016). Development of the gut microbiota and mucosal IgA responses in twins and gnotobiotic mice. Nature.

[4] Kim YG, Sakamoto K, Seo SU, Pickard JM, Gillilland MG, Pudlo NA (2017). Neonatal acquisition of Clostridia species protects against colonization by bacterial pathogens. Science.

[5] Beaumont M, Paes C, Mussard E, Knudsen C, Cauquil L, Aymard P (2020). Gut microbiota derived metabolites contribute to intestinal barrier maturation at the suckling-to-weaning transition. Gut Microbes.

[6] Healy DB, Ryan CA, Ross RP, Stanton C, Dempsey EM (2022). Clinical implications of preterm infant gut microbiome development. Nat Microbiol.

[7] DeWeerdt S. How baby’s first microbes could be crucial to future health. Nature. 2018;555(7695):S18–9. 10.1038/d41586-018-02480-6 29517013

[8] Feehley T, Plunkett CH, Bao R, Choi Hong SM, Culleen E, Belda-Ferre P (2019). Healthy infants harbor intestinal bacteria that protect against food allergy. Nat Med.

[9] Roth GA, Mensah GA, Johnson CO, Addolorato G, Ammirati E, Baddour LM (2020). Global burden of cardiovascular diseases and risk factors, 1990–2019: Update From the GBD 2019 Study. J Am Coll Cardiol.

[10] Dolk H, Loane M, Garne E, European Surveillance of Congenital Anomalies Working G (2011). Congenital heart defects in Europe: prevalence and perinatal mortality, 2000 to 2005mortality. Circulation.

[11] Oster ME, Lee KA, Honein MA, Riehle-Colarusso T, Shin M, Correa A (2013). Temporal trends in survival among infants with critical congenital heart defects. Pediatrics.

[12] Sen AC, Morrow DF, Balachandran R, Du X, Gauvreau K, Jagannath BR, et al. Postoperative Infection in Developing World Congenital Heart Surgery Programs: Data From the International Quality Improvement Collaborative. Circ Cardiovasc Qual Outcomes. 2017;10(4). Accession Number: 28408715. 10.1161/CIRCOUTCOMES.116.002935. https://www.ncbi.nlm.nih.gov/pubmed/28408715. 10.1161/CIRCOUTCOMES.116.002935 28408715

[13] Lequier LL, Nikaidoh H, Leonard SR, Bokovoy JL, White ML, Scannon PJ (2000). Preoperative and postoperative endotoxemia in children with congenital heart disease. Chest.

[14] Radman M, Mack R, Barnoya J, Castaneda A, Rosales M, Azakie A (2014). The effect of preoperative nutritional status on postoperative outcomes in children undergoing surgery for congenital heart defects in San Francisco (UCSF) and Guatemala City (UNICAR). J Thorac Cardiovasc Surg.

[15] Hoffman JI, Kaplan S (2002). The incidence of congenital heart disease. J Am Coll Cardiol.

[16] Xing J, Ying Y, Mao C, Liu Y, Wang T, Zhao Q (2018). Hypoxia induces senescence of bone marrow mesenchymal stem cells via altered gut microbiota. Nat Commun.

[17] Olney RS, Ailes EC, Sontag MK (2015). Detection of critical congenital heart defects: Review of contributions from prenatal and newborn screening. Semin Perinatol.

[18] Hyatt D, Chen GL, Locascio PF, Land ML, Larimer FW, Hauser LJ (2010). Prodigal: prokaryotic gene recognition and translation initiation site identification. BMC Bioinformatics.

[19] Fu L, Niu B, Zhu Z, Wu S, Li W (2012). CD-HIT: accelerated for clustering the next-generation sequencing data. Bioinformatics.

[20] Liu F, Smith AD, Solano-Aguilar G, Wang TTY, Pham Q, Beshah E (2020). Mechanistic insights into the attenuation of intestinal inflammation and modulation of the gut microbiome by krill oil using in vitro and in vivo models. Microbiome.

[21] Smith CA, Want EJ, O’Maille G, Abagyan R, Siuzdak G. XCMS: processing mass spectrometry data for metabolite profiling using nonlinear peak alignment, matching, and identification. Anal Chem. 2006;78(3):779–87. 10.1021/ac051437y 16448051

[22] Zeevi D, Korem T, Godneva A, Bar N, Kurilshikov A, Lotan-Pompan M (2019). Structural variation in the gut microbiome associates with host health. Nature.

[23] Santos-Medellin C, Zinke LA, Ter Horst AM, Gelardi DL, Parikh SJ, Emerson JB (2021). Viromes outperform total metagenomes in revealing the spatiotemporal patterns of agricultural soil viral communities. ISME J.

[24] Camarillo-Guerrero LF, Almeida A, Rangel-Pineros G, Finn RD, Lawley TD (2021). Massive expansion of human gut bacteriophage diversity. Cell.

[25] Nayfach S, Paez-Espino D, Call L, Low SJ, Sberro H, Ivanova NN (2021). Metagenomic compendium of 189,680 DNA viruses from the human gut microbiome. Nat Microbiol.

[26] Paez-Espino D, Chen IA, Palaniappan K, Ratner A, Chu K, Szeto E (2017). IMG/VR: a database of cultured and uncultured DNA Viruses and retroviruses. Nucleic Acids Res.

[27] Pickett BE, Sadat EL, Zhang Y, Noronha JM, Squires RB, Hunt V (2012). ViPR: an open bioinformatics database and analysis resource for virology research. Nucleic Acids Res.

[28] Fujimoto K, Kimura Y, Shimohigoshi M, Satoh T, Sato S, Tremmel G (2020). Metagenome data on intestinal phage-bacteria associations aids the development of phage therapy against pathobionts. Cell Host Microbe.

[29] Fujimoto K, Kimura Y, Allegretti JR, Yamamoto M, Zhang YZ, Katayama K (2021). Functional restoration of bacteriomes and viromes by fecal microbiota transplantation. Gastroenterology.

[30] Roux S, Hallam SJ, Woyke T, Sullivan MB. Viral dark matter and virus-host interactions resolved from publicly available microbial genomes. Elife 2015;4. Accession Number: 26200428. 10.7554/eLife.08490. https://www.ncbi.nlm.nih.gov/pubmed/26200428. 10.7554/eLife.08490 PMC4533152 26200428

[31] Hyatt D, LoCascio PF, Hauser LJ, Uberbacher EC (2012). Gene and translation initiation site prediction in metagenomic sequences. Bioinformatics.

[32] Finn RD, Clements J, Eddy SR (2011). HMMER web server: interactive sequence similarity searching. Nucleic Acids Res.

[33] Finn RD, Coggill P, Eberhardt RY, Eddy SR, Mistry J, Mitchell AL (2016). The Pfam protein families database: towards a more sustainable future. Nucleic Acids Res.

[34] Chen L, Zheng D, Liu B, Yang J, Jin Q (2016). VFDB 2016: hierarchical and refined dataset for big data analysis–10 years on. Nucleic Acids Res.

[35] Alcock BP, Raphenya AR, Lau TTY, Tsang KK, Bouchard M, Edalatmand A (2020). CARD 2020: antibiotic resistome surveillance with the comprehensive antibiotic resistance database. Nucleic Acids Res.

[36] Biswas A, Staals RH, Morales SE, Fineran PC, Brown CM (2016). CRISPRDetect: a flexible algorithm to define CRISPR arrays. BMC Genomics.

[37] Arumugam M, Raes J, Pelletier E, Le Paslier D, Yamada T, Mende DR (2011). Enterotypes of the human gut microbiome. Nature.

[38] Obeng N, Pratama AA, Elsas JDV (2016). The significance of mutualistic phages for bacterial ecology and evolution. Trends Microbiol.

[39] Wang B, Wu L, Chen J, Dong L, Chen C, Wen Z (2021). Metabolism pathways of arachidonic acids: mechanisms and potential therapeutic targets. Signal Transduct Target Ther.

[40] Nielsen OH, Ahnfelt-Ronne I, Elmgreen J (1987). Abnormal metabolism of arachidonic acid in chronic inflammatory bowel disease: enhanced release of leucotriene B4 from activated neutrophils. Gut.

[41] Robertson RC, Seira Oriach C, Murphy K, Moloney GM, Cryan JF, Dinan TG (2017). Omega-3 polyunsaturated fatty acids critically regulate behaviour and gut microbiota development in adolescence and adulthood. Brain Behav Immun.

[42] Peng M, Biswas D (2017). Short chain and polyunsaturated fatty acids in host gut health and foodborne bacterial pathogen inhibition. Crit Rev Food Sci Nutr.

[43] Rousseaux A, Brosseau C, Le Gall S, Piloquet H, Barbarot S, Bodinier M (2021). Human milk oligosaccharides: their effects on the host and their potential as therapeutic agents. Front Immunol.

[44] Walsh C, Lane JA, van Sinderen D, Hickey RM (2020). Human milk oligosaccharides: shaping the infant gut microbiota and supporting health. J Funct Foods.

[45] Laursen MF, Sakanaka M, von Burg N, Morbe U, Andersen D, Moll JM (2021). Bifidobacterium species associated with breastfeeding produce aromatic lactic acids in the infant gut. Nat Microbiol.

[46] van der Hee B, Wells JM (2021). Microbial regulation of host physiology by short-chain fatty acids. Trends Microbiol.

[47] Abrescia P, Treppiccione L, Rossi M, Bergamo P (2020). Modulatory role of dietary polyunsaturated fatty acids in Nrf2-mediated redox homeostasis. Prog Lipid Res.

[48] Daiber A, Hahad O, Andreadou I, Steven S, Daub S, Munzel T (2021). Redox-related biomarkers in human cardiovascular disease - classical footprints and beyond. Redox Biol.

[49] Soyyilmaz B, Miks MH, Rohrig CH, Matwiejuk M, Meszaros-Matwiejuk A, Vigsnaes LK. The mean of milk: a review of human milk oligosaccharide concentrations throughout lactation. Nutrients. 2021;13(8). Accession Number: 34444897. 10.3390/nu13082737. https://www.ncbi.nlm.nih.gov/pubmed/34444897. 10.3390/nu13082737 PMC8398195 34444897

[50] Grootjans J, Thuijls G, Verdam F, Derikx JP, Lenaerts K, Buurman WA (2010). Non-invasive assessment of barrier integrity and function of the human gut. World J Gastrointest Surg.

[51] Pathan N, Burmester M, Adamovic T, Berk M, Ng KW, Betts H (2011). Intestinal injury and endotoxemia in children undergoing surgery for congenital heart disease. Am J Respir Crit Care Med.

[52] Cheng CH, Lin PT, Liaw YP, Ho CC, Tsai TP, Chou MC, et al. Plasma pyridoxal 5’-phosphate and high-sensitivity C-reactive protein are independently associated with an increased risk of coronary artery disease. Nutrition. 2008;24(3):239–44. 10.1016/j.nut.2007.12.003 18312786

[53] Nagpal R, Tsuji H, Takahashi T, Nomoto K, Kawashima K, Nagata S, et al. Ontogenesis of the gut microbiota composition in healthy, full-term, vaginally born and breast-fed infants over the first 3 years of life: a quantitative bird’s-eye view. Front Microbiol. 2017;8:1388. 10.3389/fmicb.2017.01388 PMC5519616 28785253

[54] Morelli L (2008). Postnatal development of intestinal microflora as influenced by infant nutrition. J Nutr.

[55] Shao Y, Forster SC, Tsaliki E, Vervier K, Strang A, Simpson N (2019). Stunted microbiota and opportunistic pathogen colonization in caesarean-section birth. Nature.

[56] Ho NT, Li F, Lee-Sarwar KA, Tun HM, Brown BP, Pannaraj PS (2018). Meta-analysis of effects of exclusive breastfeeding on infant gut microbiota across populations. Nat Commun.

[57] Penders J, Thijs C, Vink C, Stelma FF, Snijders B, Kummeling I (2006). Factors influencing the composition of the intestinal microbiota in early infancy. Pediatrics.

[58] Li K, Zhu Q, Jiang F, Li H, Liu J, Yu T (2022). Monitoring microbial communities in intensive care units over one year in China. Sci Total Environ.

[59] Milani C, Duranti S, Bottacini F, Casey E, Turroni F, Mahony J, et al. The first microbial colonizers of the human gut: composition, activities, and health implications of the infant gut microbiota. Microbiol Mol Biol Rev 2017;81(4). Accession Number: 29118049. 10.1128/MMBR.00036-17. https://www.ncbi.nlm.nih.gov/pubmed/29118049. 10.1128/MMBR.00036-17 PMC5706746 29118049

[60] Henrick BM, Chew S, Casaburi G, Brown HK, Frese SA, Zhou Y (2019). Colonization by B. infantis EVC001 modulates enteric inflammation in exclusively breastfed infants. Pediatr Res.

[61] Van den Abbeele P, Sprenger N, Ghyselinck J, Marsaux B, Marzorati M, Rochat F. A comparison of the in vitro effects of 2'fucosyllactose and lactose on the composition and activity of gut microbiota from infants and toddlers. Nutrients. 2021;13(3). Accession Number: 33668823. 10.3390/nu13030726. https://www.ncbi.nlm.nih.gov/pubmed/33668823. 10.3390/nu13030726 PMC7996240 33668823

[62] Zhang Z, Tang H, Chen P, Xie H, Tao Y (2019). Demystifying the manipulation of host immunity, metabolism, and extraintestinal tumors by the gut microbiome. Signal Transduct Target Ther.

[63] Meng D, Sommella E, Salviati E, Campiglia P, Ganguli K, Djebali K (2020). Indole-3-lactic acid, a metabolite of tryptophan, secreted by Bifidobacterium longum subspecies infantis is anti-inflammatory in the immature intestine. Pediatr Res.

[64] Gasaly N, de Vos P, Hermoso MA (2021). Impact of bacterial metabolites on gut barrier function and host immunity: a focus on bacterial metabolism and its relevance for intestinal inflammation. Front Immunol.

[65] Jeurink PV, van Esch BC, Rijnierse A, Garssen J, Knippels LM (2013). Mechanisms underlying immune effects of dietary oligosaccharides. Am J Clin Nutr.

[66] Stein-Thoeringer CK, Nichols KB, Lazrak A, Docampo MD, Slingerland AE, Slingerland JB (2019). Lactose drives Enterococcus expansion to promote graft-versus-host disease. Science.

[67] Llorente C, Jepsen P, Inamine T, Wang L, Bluemel S, Wang HJ (2017). Gastric acid suppression promotes alcoholic liver disease by inducing overgrowth of intestinal Enterococcus. Nat Commun.

[68] Steck N, Hoffmann M, Sava IG, Kim SC, Hahne H, Tonkonogy SL (2011). Enterococcus faecalis metalloprotease compromises epithelial barrier and contributes to intestinal inflammation. Gastroenterology.

[69] Manfredo Vieira S, Hiltensperger M, Kumar V, Zegarra-Ruiz D, Dehner C, Khan N (2018). Translocation of a gut pathobiont drives autoimmunity in mice and humans. Science.

[70] Chen L, Wang D, Garmaeva S, Kurilshikov A, Vich Vila A, Gacesa R (2021). The long-term genetic stability and individual specificity of the human gut microbiome. Cell.

[71] Wang D, Doestzada M, Chen L, Andreu-Sanchez S, van den Munckhof ICL, Augustijn HE (2021). Characterization of gut microbial structural variations as determinants of human bile acid metabolism. Cell Host Microbe.

[72] Peters-Golden M, Canetti C, Mancuso P, Coffey MJ (2005). Leukotrienes: underappreciated mediators of innate immune responses. J Immunol.

[73] Liang G, Zhao C, Zhang H, Mattei L, Sherrill-Mix S, Bittinger K (2020). The stepwise assembly of the neonatal virome is modulated by breastfeeding. Nature.

[74] Rodriguez-Valera F, Martin-Cuadrado AB, Rodriguez-Brito B, Pasic L, Thingstad TF, Rohwer F (2009). Explaining microbial population genomics through phage predation. Nat Rev Microbiol.

[75] Knowles B, Silveira CB, Bailey BA, Barott K, Cantu VA, Cobian-Guemes AG (2016). Lytic to temperate switching of viral communities. Nature.

[76] Lopez CA, Winter SE, Rivera-Chavez F, Xavier MN, Poon V, Nuccio SP, et al. Phage-mediated acquisition of a type III secreted effector protein boosts growth of salmonella by nitrate respiration. mBio. 2012;3(3). Accession Number: 22691391. 10.1128/mBio.00143-12. https://www.ncbi.nlm.nih.gov/pubmed/22691391. 10.1128/mBio.00143-12 PMC3374392 22691391

[77] Edlin G, Lin L, Kudrna R (1975). Lambda lysogens of E. coli reproduce more rapidly than non-lysogens. Nature.

[78] Brussow H, Canchaya C, Hardt WD. Phages and the evolution of bacterial pathogens: from genomic rearrangements to lysogenic conversion. Microbiol Mol Biol Rev 2004;68(3):560–602, table of contents 10.1128/MMBR.68.3.560-602.2004 PMC515249 15353570

[79] Nedialkova LP, Sidstedt M, Koeppel MB, Spriewald S, Ring D, Gerlach RG (2016). Temperate phages promote colicin-dependent fitness of Salmonella enterica serovar Typhimurium. Environ Microbiol.

[80] Secor PR, Sweere JM, Michaels LA, Malkovskiy AV, Lazzareschi D, Katznelson E (2015). Filamentous bacteriophage promote biofilm assembly and function. Cell Host Microbe.

