# Supplementary material for: Mapping the early life gut microbiome in neonates with critical congenital heart disease: multiomics insights and implications for host metabolic and immunological health
Source: Microbiome. 2022 Dec 30;10:245. doi: 10.1186/s40168-022-01437-2 (PMC9801562; doi:10.1186/s40168-022-01437-2)
Supplement: Supplementary file 2 — Additional file 1: Supplementary file 1. [file 40168_2022_1437_MOESM1_ESM.pdf]

Supplementary Materials

Mapping the early life gut microbiome in neonates with critical congenital heart disease: multiomics insights and implications for host metabolic and immunological health.

This PDF file includes:

Methods

Supplementary Table S1 to S4

Table S1. Subject cohort demographics.

Table S2. Patients with adverse outcomes.

Table S3. Demographic and Clinical Characteristics of CCHD Patients Diagnosed with a good and poor prognosis.

Table S4. Univariable and multivariable logistic regression analyses of potential risk factors for surgical prognosis in the study cohort.

Supplementary Fig. S1 to S16

Figure S1. Flowchart for study cohort enrollment.

Figure S2. Gut virome alterations in CCHD associated with bacterial profile.

Figure S3. Functional genes associated with virulence factors and antibiotic resistance in *Siphoviridae* targeting *Enterococcus* in CCHD group.

Figure S4. Differences in fecal metabolome between CCHD and HC.

Figure S5. A co-occurrence network deduced from the differential bacterial species and fecal metabolites identified in CCHD versus HC.

Figure S6. Alterations in microbial functionality between CCHD and HC.

Figure S7. Overview of structural variation profile in gut microbiome in the study cohort.

Figure S8 The abundance of human milk oligosaccharide (HMO)-utilization genes associated with fecal level of 2-fucosyllactose.

Figure S9. Association between microbial phenylalanine, tyrosine and tryptophan biosynthesis pathway and indolelactic acid.

Figure S10. Differences in gut bacterial composition in pairwise comparisons of CCHD-P *versus* HC and CCHD-G *versus* HC.

Figure S11. Comparison of serum inflammatory biomarkers between CCHD-G and CCHD-P subgroups.

Figure S12. Comparison of intestinal permeability biomarkers between CCHD-G and CCHD-P subgroups.

Figure S13. ROC curve showing discrimination accuracy of the predictive model for prognostic stratification in the study cohort.

Figure S14. Differences in fecal metabolome between CCHD-P and CCHD-G.

Figure S15. A co-occurrence network deduced from the differential bacterial species and fecal metabolites identified in CCHD-P versus CCHD-G.

Figure S16. Conceptual diagram of potential mechanisms linking the gut microbiome, microbial metabolites and host pathological phenotypes including gut barrier impairment and systemic inflammatory response.

Methods

Participants recruitment and samples collection

The current study was approved by the Human Research and Ethics Committee of Fuwai Hospital Chinese Academy of Medical Sciences (Approval NO. 2019-1300) and Shanghai Children's Medical Center (Approval NO. SCMCIRB-K2021021) in accordance with the Declaration of Helsinki conventions on human studies, and written informed consent was obtained from each participant. For patient cohort recuitment, inclusion criteria were full-term neonates diagnosed with critical congenital heart diease (CCHD).[1] Exclusion criteria included (i) diagnosed with any extra-cardiac medical condition which could affect metabolism, nutritional status or physical health, including gastrointestinal symptoms, infectious diseases, chromosomal syndromes, hepatorenal dysfunction, severe malnutrition, etc; (ii) previous usage of probiotics, antibiotics or immunosuppressant which could potentially affect gut microbial composition. Briefly, as shown in Figure S1, 116 neonates with CCHD were enrolled between December 2019 and August 2021. Biological samples (including blood samples and stool samples) and clinical parameters during hospitalization were collected from 96 CCHD patients that met the inclusion and exclusion criteria. Participants that did not obtain qualified stool samples for multiomics analysis or missed demographic information for comparable analysis were further excluded in the subsequent analyses. As a result, 19 well-sampled cases of CCHD with poor surgical prognosis and 26

matched CCHD controls with good surgical prognosis were finally included for multi-omics analysis. In addition, we simultaneously recruited 75 healthy full-term neonates as healthy controls (HCs), with written informed consent obtained from their parents. Finally, 50 matched HCs based on demographic information were included for subsequent comparable analysis, from whom stool samples were collected. All biological samples were couriered to the laboratory in insulated envelopes containing frozen packs and stored at -80℃ until analysis.

Phenotype data

Phenotypic data assessed in the present study included baseline demographics (e.g., age, gender, birth weight) and clinical characteristics (e.g., main diagnosis, perioperative data, adverse surgical outcomes). Specifically, the baseline demographic information of neonates with CCHD and matched HCs was summarized in Supplementary Table S1. As for prognostic stratification of patient cohort, in alignment with previous research,[2] composite adverse events were used to define the poor prognosis (summarized in Table S2), including in-hospital death, extracorporeal membrane oxygenation (ECMO) placement, length of postoperative hospital stay (LOPS) > 30 days, and other severe postoperative complications associated with cardiopulmonary system, acute kidney injury and systemic inflammation. In total, 19 patients with one or more adverse event/s listed above were classified as having a poor prognosis. The comparison of clinical characteristics between CCHD patients with poor prognosis (termed CCHD-P) and their peers with good prognosis (termed CCHD-G) was summarized in Table S3.

Bioinformatic analyses

Shotgun metagenomic analysis of fecal samples

Microbial DNA was isolated from fecal samples of all subjects and sequenced as previously described following the similar protocol.[3] In brief, total genomic DNA was extracted using QIAamp PowerFecal Pro DNA Kit (QIAGEN, USA) according to the manufacturer's instructions. Concentration and purity of extracted DNA were determined with TBS-380 and NanoDrop2000, respectively. DNA extract quality was checked on 1% agarose gel. High

quality DNA was fragmented using the Covaris M220 for library construction. Paired-end library was constructed using NEXTFLEX Rapid DNA-Seq (Bioo Scientific, Austin, TX, USA). Sequencing was performed on Illumina NovaSeq (Illumina Inc., San Diego, CA, USA) at Majorbio Bio-Pharm Technology Co., Ltd. (Shanghai, China).

Overall, the shotgun metagenomic sequencing generated a total of 11.12 billion raw reads across all fecal samples (N=95), with an average read count of 117.07 million per sample. Fastp (https://github.com/OpenGene/fastp, version 0.20.0) was utilized to remove the adaptors and low-quality reads.[4] After quality filtering and trimming, 114.04 million clean reads were obtained on average (97.7% of the total raw reads), producing an average sequencing size of 17 GB (Gigabases) per sample. Then the clean reads were aligned to the human genome assembly (GRCh38.p13, http://asia.ensembl.org/Homo_sapiens/Info/Index) by using the Burrows-Wheeler Aligner (BWA)[5] (http://bio-bwa.sourceforge.net) and any hit associated with the reads and their mated reads were removed. The high quality non-host reads of each fecal sample were assembled using MEGAHIT[6] (https://github.com/voutcn/megahit, version 1.1.2) and contigs shorter than 300bp were discarded. Open reading frames (ORFs) from each contig were predicted using Prodigal (version 2.6.3).[7] Only ORF longer than 100bp was considered in downstream analyses. Gene sequences were clustered into a non-redundant gene catalogue using CD-HIT(version 4.6)[8] at 95% identity and 90% coverage. The high quality reads were mapped to the non-redundant gene catalogue with 95% identity using SOAPaligner (http://soap.genomics.org.cn/, version 2.21).[9]

Gene sets were annotated to the NCBI NR database with an e-value (cutoff = 10^{-5}) using Diamond (version 2.0.11) to obtain taxonomic assignment of bacteria. Using the alignment results, each gene is assigned to the highest scoring taxonomy based on the same database. In order to obtain the KEGG annotation, the gene sets were aligned with KEGG database (Kyoto Encyclopedia of Genes and Genomes, http://www.genome.jp/kegg/)[10] using DIAMOND with an e value cutoff of 10^{-5}. The microbiome diversity analyses (including alpha and beta diversity) were conducted and visualized using the vegan and ggplot2 packages in R (version 4.0.2). Specifically, alpha diversity metrics (including Chao and Shannon indexes) were calculated at the phylum, family, genus, and species levels. The differences in alpha diversity

indexes between groups were tested by Wilcoxon rank sum test. As for beta diversity analysis, principal coordinate analysis (PCoA) based on Bray-Curtis (BC) distances was performed to visually evaluate the overall difference and similarity of bacterial communities and KEGG orthology between different groups (https://doi.org/10.1038/s41591-019-0533-0). Differences in PCoA ordination between groups were tested by permutational multivariate analysis of variance (PERMANOVA). The discriminative bacterial species and KEGG orthology between the two groups were identified using Linear discriminant analysis Effect Size (LEfSe) with a linear discriminant analysis (LDA) score > 2.0.

Untargeted metabolomics profiling of fecal samples

Untargeted metabolomics analysis of fecal samples was conducted as previously described.[11] Briefly, individual samples were accurately weighed. 50 mg of sample was weighted to an EP tube, and 1000 μL extract solution (methanol: acetonitrile: water = 2: 2: 1, with isotopically-labelled internal standard mixture) was added. Then the samples were homogenized at 35 Hz for 4 min and sonicated for 5 min in ice-water bath. The homogenization and sonication cycle was repeated for 3 times. Then the samples were incubated for 1 h at -40 °C and centrifuged at 12000 rpm for 15 min at 4 °C. The resulting supernatant was transferred to a fresh glass vial for analysis. The quality control (QC) sample was prepared by mixing an equal aliquot of the supernatants from all of the samples. Processed samples were characterized by an UHPLC system (Vanquish, Thermo Fisher Scientific) with a UPLC BEH Amide column (2.1 mm × 100 mm, 1.7 μm) coupled to Q Exactive HFX mass spectrometer (Orbitrap MS, Thermo). The raw data were converted to the mzXML format using ProteoWizard and processed with an in-house program, which was developed using R based on the R package xcms (Anal Chem. 2006 Feb 1;78(3):779-87), for peak detection, extraction, alignment, and integration. Then an in-house MS2 database (BiotreeDB), which was built on multiple open source databases, namely HMDB (https://hmdb.ca/), MONA (https://mona.fiehnlab.ucdavis.edu/), and METLIN (https://metlin.scripps.edu/), in combination with our self-constructed assembly of mass spectra derived from more than 3000 standards, was applied in metabolite annotation. The cutoff for annotation was set at 0.3. The statistics function prcomp R-package

(www.r-project.org) was used to carry out the supervised multiple regression orthogonal partial least-squares discriminant analysis (OPLS-DA). In order to avoid overfitting, a permutation test (200 permutations) was performed. A threshold variable importance in the projection (VIP > 1) obtained from the OPLS-DA model and the Student's t test ($P < 0.05$) were used to assess the significance of difference in abundance of metabolites. Normalized data were analyzed using a modified t test and the value of variable importance in the projection (VIP) of the first principal component in OPLS-DA analysis was obtained to identify metabolites that may differ significantly among experimental groups. In addition, commercial databases including KEGG (http://www.genome.jp/kegg/) and MetaboAnalyst (http://www.metaboanalyst.ca/) were used for pathway enrichment analysis.

Targeted metabolites measurement of short chain fatty acids (SCFAs)

The SCFA concentrations were determined as previously described.[12] Briefly, take 50 mg fecal sample into 2 mL EP tubes, extracted with 1.0 mL dH_2O, vortex mixing for 10 s. Ho-mogenized in ball mill for 4 min at 40 Hz, then ultrasound treated for 5 min (incubated in ice water). Centrifuge for 20 min at 5000 rpm, 4°C. Transfer the supernatant (0.8 mL) into a fresh 2 mL EP tubes, add 0.10 mL 50% H_2SO_4 and 0.8 mL of 2-Methylvaleric acid (25 mg/mL stock in ethyl ether) as internal standard. Centrifuge for 10 min at 12000 rpm, 4°C. Keep at -20°C for 30 min. Transfer the supernatant into a fresh 2 mL glass vial for GC-MS analysis. GC-MS analysis was performed using an Shimadzu GC-2030 gas chromatograph system coupled with an Shimadzu QP2020 NX mass spectrometer. The system utilized a HP-FFAP capillary column. A 1 uL aliquot of the analyte was injected in split mode (5:1). Helium was used as the carrier gas, the front inlet purge flow was 3 mL/min, and the gas flow rate through the column was 1 mL /min. The initial temperature was kept at 80 °C for 1 min, then raised to 200 °C at a rate of 10 °C/min for 5 min, then kept for 1 min at 240 °C at a rate of 40 °C/min. The injection, transfer line, quad and ion source temperatures were 240 °C, 240 °C, 200 °C and 150 °C respectively. The energy was -70 eV in electron impact mode. The mass spectrometry data were acquired in full-scan mode with the m/z range of 33-150 after a solvent delay of 3.5 min.

Analysis of serum proinflammatory cytokines and intestinal permeability biomarkers

The blood samples were centrifuged at 3,000 rpm for 10 min and the supernatants were collected and stored at -80°C for subsequent analysis. The Luminex Human Discovery multiplex assay kit (Catalog Number: LXSAHM; R&D Systems, Inc, USA) was used for simultaneous measurement of cytokines IL-1β, IL-6, IL-8, TNF-α and interferon (INF)-γ in serum samples. Upon completion of each multiplex assay, the amounts of serum cytokines were analyzed via Luminex. In addition, a panel of serum biomarkers of intestinal permeability was used to comprehensively investigate the gut barrier function, namely zonulin, D-lactate (D-LA), intestinal fatty acid binding protein (iFABP), lipopolysaccharide (LPS), and lipopolysaccharide binding protein (LBP). Specifically, zonulin is a modulator of intestinal permeability, with the increased serum level indicating a breakdown of intestinal epithelial tight junctions[13]; D-LA is a fermentation product produced by commensal bacteria residing in the intestinal lumen, with very low circulating level being detected normally, but in case of gut barrier impairment, the serum level of D-LA would rise as a consequence of increased translocation across the intestinal mucosa [14, 15]; iFABP is a cytosolic protein exclusively present in intestinal epithelial cells and is easily released into the bloodstream when the intestinal epithelium is disrupted[16]; LPS is a bacterial component that normally presents in the intestinal lumen and increased serum level of this molecule could be considered as a sign of bacterial translocation as well as impaired gut barrier function[17]; LBP is an acute-phase circulating protein that could specifically bind to LPS and is therefore proposed as a serum biomarker of intestinal permeability indicating increased transepithelial uptake of LPS [18, 19], which could also circumvent the pitfall of LPS-contamination of common lab equipment. The commercially available ELISA kits (Bio-swamp Life Science, Wuhan, China) were used to measure these intestinal permeability biomarkers according to manufactures' instructions.

Characterization of microbial genetic structural variations

Structural variants (SVs) are highly variable segments of bacterial genomes that are deleted from some individuals (deletion SVs, dSVs) or present in a variable number of copies (variable SVs, vSVs) in others. Based on the cleaned non-host reads, we applied the

SGV-Finder pipeline[20] to classify the microbial SVs (including dSVs and vSVs) of all 95 samples from CCHD and HC groups. The SV-screening procedure is mainly divided into two steps: (1) run ICRA, which is an 'iterative coverage-based read assignment' algorithm, to resolve ambiguous read assignments to regions that are similar between different bacteria, by using information on bacterial relative abundances in the microbiome, their genomic sequencing coverage, and sequencing and alignment qualities, fine map the metagenomic reads to avoid ambiguous alignment (https://doi.org/10.1038/s41586-019-1065-y). (2) run SGV-Finder, which analyses coverage depth across all microbial genomes in all samples to characterize SVs with respect to the standardized coverage of a genome in a given sample (https://doi.org/10.1038/s41586-019-1065-y). Finally, the distribution of dSV and vSV in our sample is obtained respectively. Furthermore, The variability of microbial SVs between individuals were determined by Canberra distance.

Virome analysis based on metagenomic sequencing data

1. **Identification of viral contigs:** with deep sequencing metagenomic data, we identified putative viral contigs from the bulk metagenomes following contig assembly. DeepVirFinder was used to detect viral contigs[21], which is based on deep learning method to identify and screen contigs composed of viral moieties. This method takes advantage of deep learning and big data without the need to align with reference sequences, which significantly improves the speed and accuracy of virus identification. The default prediction model was used to identify viral contigs, and only predicted contigs with a length greater than 1000bp were extracted. Then, contigs with P value < 0.05 and score ≥ 0.7 were selected as candidate virus contigs for subsequent analysis.

2. **Viral taxonomic profiling:** to ensure the accuracy of viral taxonomic annotation, five latest improved databases were employed to classify taxonomy of putative viral contigs: (1) viral RefSeq genomes database (virus reference genome sequences downloaded from NCBI, containing 11,598 reference genomes) (https://www.ncbi.nlm.nih.gov/labs/virus/vssi/#/virus?SeqType_s=Genome); (2) Gut Phage Database (GPD).[22] This database contains a collection of ~142,000 non-redundant viral genomes (>10 kb) by mining a dataset containing 28,060 globally distributed human gut

metagenomes and 2,898 reference genomes of cultured gut bacteria; (3) Metagenomic Gut Virus (MGV) catalogue,[23] which contains a large number of subgenomes from 11,810 human stool samples from 61 previously published studies; (4) IMG/VR database (version: IMG_VR_2020-10-12_5.1 - IMG/VR v3),[24] which is the largest publicly available database of 3908 isolate reference DNA viruses with 264,413 computationally identified viral contigs from >6,000 ecologically diverse metagenomic samples; (5) Virus Pathogen Resource (ViPR, https://www.viprbrc.org/brc/home.spg?decorator=vipr),[25] which integrates data from external sources (GenBank, UniProt, Immune Epitope Database, Protein Data Bank, etc.). Technically, the viral contig sequences were first searched against the viral RefSeq genomes database using DIAMOND with an E value <10^{-5}, and select the most similar result of each sequence alignment as virus classification information. If the viral contig is not aligned taxonomy to the viral RefSeq genomes database, the other four databases were then used to perform the same standard alignment procedure, and select the database alignment result with the highest identity and the most complete virus classification information as the final virus classification information. After removing the contigs that were not aligned with the above five databases, a total of 22,245 virus annotation were finally identified, which were used as the basis for subsequent analysis of virus.

3. **Prediction of phage lifestyles:** PHACTS was used to classify phage lifestyles (i.e. temperate or lytic bacteriophages) of putative viral contigs. In specific, Of 22,245 viral contigs, 22,174 were classified as “Bacteriophages” and used for PHACTS analysis. Ten replicate PHACTS predictions were performed. Probability values obtained from PHACTS were standardized between -1 and 1, which was presented as probability of “Lytic” or “Temperate”.

Prophage-based bacteria-phage association analysis

Prophage-based bacteria-phage association analysis was conducted in alignment with previous research.[26, 27] In brief, the prophage sequences in bacterial contigs were predicted using VirSorter (version 1.0.3).[28] Initially, the bacterial contigs (≥ 5 kb) from all samples were analyzed by VirSorter using both RefSeqABVir (-db 1) and Viromes (-db 2). Then the predicted prophage sequences of VirSorter categories 4 or 5 (presence of viral hallmark genes

or enrichment of viral-like genes in a prophage region) were extracted. The positions of the predicted prophage sequences on bacterial contigs were determined through megablast (BLAST+ version 2.7.1) searches (E value < 10^{-100} and ≥ 95% identity), and prophage sequences were merged if their positions overlapped. Last, prophage sequences longer than 3 kb were extracted and listed as final prophage sequences.

Gene annotation of viral contigs was based on open reading frames (ORFs). We first predicted the ORFs on the contigs by using MetaProdigal (version 2.6.3).[29] The identified ORFs were then queried by hmmscan in HMMER3 (version 3.3.2)[30] against the PfamA database (version 34.0)[31] with an E value < 10^{-5}, and were assigned to related gene categories with a customized Pfam-category annotation table.[26] Similar procedure was applied to annotate the gene function against the virulence factor database (VFDB) [32] and comprehensive antibiotic resistance database (CARD).[33]

CRISPR-based bacteria-phage association analysis

The CRISPR-based bacteria-phage association analysis was conducted in alignment with previous research.[26, 27] In brief, we first identified CRISPR spacers on bacterial contigs (≥ 5 kb) by using the CRISPR array identification program CRISPRDetect.[34] To identify the target phages of the CRISPR spacers, we then queried the identified spacers using blastn (BLAST+ version 2.12.0) (https://doi.org/10.1186/1471-2105-10-421) against the viral contigs and extracted the aligned spacers with >90% of their length aligned with a minimum identity level of 95% and a maximum E value of 5×10^{-3}.

Enterotype analysis

Enterotype analysis was performed at the genus level using the Jensen-Shannon divergence (JSD) distance and the Partitioning Around Medoids (PAM) clustering algorithm in the R package cluster. The optimal number of clusters was determined by Calinski-Harabasz (CH) index using the R package clusterSim. PCoA was performed to visualize the clusters of samples using the R package ade4.[35] The top 10 most abundant species enriched in each enterotype were selected for correlation analysis. Correlations between species were determined by Spearman's rank correlation analysis. For plotting purpose, only correlations

with coefficient > +0.6 or < -0.6 were plotted in the network using Cytoscape 3.9.0.

Metagenome-wide association analysis

(1) Associations between microbial abundance, KEGG pathway abundance, microbial vSVs and fecal metabolites: Spearman's rank correlation coefficients were calculated with adjusted P values to determine the associations between continuous microbial variables and fecal metabolites.

(2) Associations between microbial dSVs and fecal metabolites: Two-sided Mann-Whitney U-test was used to calculate significance of associations between dSVs (binary variables) and the same metabolites.

Mediation analysis

To investigate the mediation linkages between multilevel microbial features (i.e., microbial abundance, KEGG pathways and SVs), fecal metabolites, and host pathological phenotypes (i.e., serum biomarkers of inflammation and intestinal permeability), we first identified microbe-metabolite-biomarker groups in which all variables significantly correlated with each other as candidate groups with a potential mediation effect. Then we performed bi-directional analysis on the candidate groups using the R package mediation (version 4.5.0).

Statistical analysis

Basic statistical analyses (including Student's T-test, Wilcoxon Test, chi-square test, and Fisher's exact test) were performed as appropriate to compare continuous and categorical variables using SPSS (version 25). Univariable and multivariable logistic regression analyses were performed to evaluate the predictive value of *Enterococcus* abundance in prognostic stratification in the study cohort (SPSS version 25). Therefore, the log10-transformed relative abundance of *Enterococcus* species (identified by LDA in the comparison of CCHD-P *versus* CCHD-G), baseline demographics, and preoperative and intraoperative variables were first tested by univariable logistic regression analysis. The variables with $P < 0.1$ in univariable logistic regression analysis or clinically relevant variables were further included in a forward selection multivariable logistic regression model. Receiver-operating characteristic curve was

used to evaluate the discriminative performance of the predictive model constructed based on multivariable logistic regression analysis. Other statistical details related to metagenomic and metabolomic data can be found in the figure legends and methods. P values of < 0.05 were considered significant.

TABLE S1. Study cohort demographics

Baseline characteristics	healthy controls (n = 50)	CCHD (n = 45)	P value
Gestational age (weeks)	39（38 – 40）	39（38 – 40）	0.981
Birth length（cm）	50（49 – 50）	49（48 – 50）	0.142
Birth weight（kg）	3.26 ± 0.32	3.20 ± 0.37	0.423
Female gender, n (%)	17 (34%)	14（31.1%）	0.764
Delivery Mode<br>Cesarean section, n (%)	28 (56%)	26（57.8%）	0.861
Age at sampling (days)	17（11 – 24）	16（10.5 – 22）	0.294
Breast milk exposure, n (%)	41 (82%)	35 (77.8%)	0.607

Notes: Data are presented as median (interquartile interval) or mean ± SD or percentage (%). Statistical significance is determined by Mann-Whitney U-test, Student's t-test, or the chi-square test as appropriate.

CCHD, critical congenital heart disease.

TABLE S2. Patients with adverse outcomes

**Adverse Outcomes**	**Number of Patients**
Death	2
ECMO placement	1
LOPS > 30 d	7
Severe complications	
Respiratory failure or extubation failure	7
Thromboembolic events	2
Significant cardiac disorders	3
Severe cerebrovascular accident	0
Severe renal failure	6
Sepsis	1
Total	19

Notes: Values are presented as number of patients. Total number exceeds 19 as patients may have had more than one major adverse outcome. ECMO, Extracorporeal Membrane Oxygenation; LOPS, length of postoperative hospital stay.

TABLE S3. Demographic and clinical characteristics of CCHD patients with good and poor prognosis

	Good prognosis (n = 26)	Poor prognosis (n = 19)	P value
Demographic data			
Gestational age (weeks)	39 (38 – 39）	39 (38 – 40）	0.394
Delivery Mode<br>Cesarean section, n (%)	16 (61.5%)	10 (52.6%)	0.550
Female gender, n (%)	8（30.8%)	6（31.6%)	0.954
Age at operation (days)	16（11 – 22.25）	15（9 – 21）	0.497
Length at operation (cm)	48.8（48 – 50）	49.5 (48.5 – 50.5)	0.260
Weight at operation (kg)	3.2 ± 0.36	3.2 ± 0.38	0.871
Breast milk exposure n (%)	19 (73.1%)	16 (84.2%)	0.481
Preoperative data			
Main diagnosis, n	TGA (11) ; TAPVC (10); PAA (4); TA (1)	TGA (8); TAPVC (8); PAA (2) ;TA (1)	—
SpO_2 (%)	60.15 (47.23 – 76.60)	58.4 (48.6 – 69.5)	0.629
RACHS-1 category n (%)			0.771
1	0	0	
2	0	0	
3	11	6	
4	14	12	
5	1	1	
ABC category n (%)			0.844
1	0	0	
2	5	2	
3	10	8	
4	11	9	
LVEF (%)	64（60 – 65）	65（60 – 65）	0.766
Intraoperative data			
CPB time (min)	128.27 ± 37.45	142 ± 35.43	0.221
ACC time (min)	81.23 ± 27.88	85.11 ± 35.23	0.683
T_{min} (℃)	29.35 ± 2.27	29.71 ± 1.87	0.584
Blood loss (ml)	20 (8.25 – 46.25)	20 (0 – 35)	0.736
Infusion volume (ml)	35.19 ± 10.63	41.05 ± 14.30	0.122

Notes: Data are presented as median (interquartile interval) or mean ± SD or percentage (%). Statistical significance is determined by Mann-Whitney U-test, Student's t-test, or the chi-square test as appropriate.

CCHD, critical congenital heart disease; TGA, transposition of the great arteries; TAPVC, total anomalous pulmonary venous connection; PAA, pulmonary artery atresia; TA, truncus arteriosus; SpO_2, blood oxygen saturation; RACHS-1, Risk Adjustment for Congenital Heart Surgery; ABC, Aristotle Basic Complexity; LVEF, left ventricular ejection fraction; CPB, cardiopulmonary bypass; ACC, aortic cross-clamp; T_{min}, the minimum temperature.

TABLE S4. Univariable and multivariable logistic regression analyses of potential risk factors for surgical prognosis in the study cohort

Variables	Univariable analysis		Multivariable analysis	
	OR (95% CI)	P value	OR (95% CI)	P value
Log_{10}(*Enterococcus faecium*)*	2.697 (1.249–5.824)	0.012	5.711 (1.883–17.322)	0.002
Log_{10}(*Enterococcus gallinarum*)*	1.691 (0.950–3.011)	0.074		
Log_{10}(*Enterococcus sp. HMSC035C10*)*	2.625 (1.210–5.691)	0.015		
Log_{10}(*Enterococcus hirae*)*	2.642 (1.142–6.114)	0.023		
Log_{10}(*Enterococcus avium*)*	2.037 (1.057–3.924)	0.033		
Log_{10}(*Enterococcus columbae*)	1.007 (0.980–1.034)	0.617		
Log_{10}(*Enterococcus saccharolyticus*)	1.016 (0.992–1.041)	0.188		
Log_{10}(*Enterococcus casseliflavus*)	1.545 (0.860–2.774)	0.145		
Log_{10}(*Enterococcus durans*)*	2.017 (1.021–3.987)	0.043		
Gestational age* (weeks)	1.293 (0.730–2.289)	0.379		
RACHS-1 category*	0.857 (0.048–15.224)	0.916		
ABC category*	0.978 (0.272–3.519)	0.973		
Age at operation* (days)	0.964 (0.875–1.062)	0.454		
Weight at operation*（kg）	0.498 (0.104–2.379)	0.382		
SpO_2* (%)	0.987 (0.946–1.030)	0.542		
LVEF* (%)	1.000 (0.942–1.063)	0.988		
CPB time* (min)	1.011 (0.994–1.028)	0.219	1.029 (1.003–1.055)	0.027
ACC time* (min)	1.004 (0.985–1.024)	0.675		
Blood loss* (ml)	0.993 (0.961–1.026)	0.668		
T_{min}* (℃)	1.086 (0.814–1.448)	0.575		
Infusion volume* (ml)	1.041 (0.989–1.095)	0.125	1.078 (1.003–1.158)	0.042

Notes: The *Enterococcus* species are identified by LDA in the comparison of CCHD-P *versus* CCHD-G, with their relative abundances log10-transformed and tested by univariable logistic regression analysis. The variables with $P < 0.1$ in univariable logistic regression analysis or clinically relevant variables (marked by *) are further included in a forward selection multivariable logistic regression model.

LDA, linear discriminant analysis; CCHD-P, CCHD patients with poor surgical prognosis; CCHD-G, CCHD patients with good surgical prognosis; RACHS-1, Risk Adjustment for Congenital Heart Surgery; ABC, Aristotle Basic Complexity; SpO_2, blood oxygen saturation; LVEF, left ventricular ejection fraction; CPB, cardiopulmonary bypass; ACC, aortic cross-clamp; T_{min}, the minimum temperature.

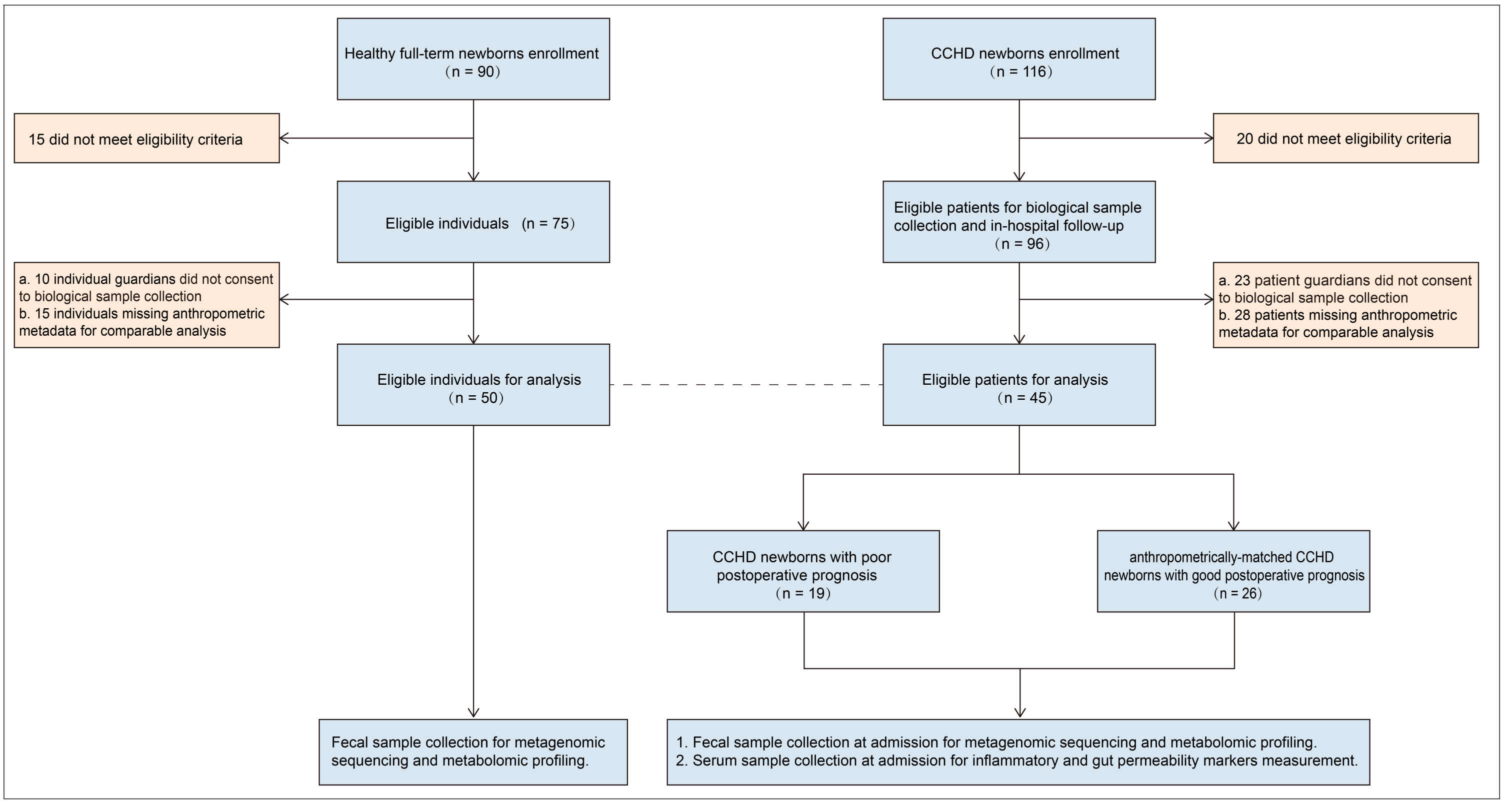

Figure S1. Flowchart for study cohort enrollment. CCHD, critical congenital heart disease.

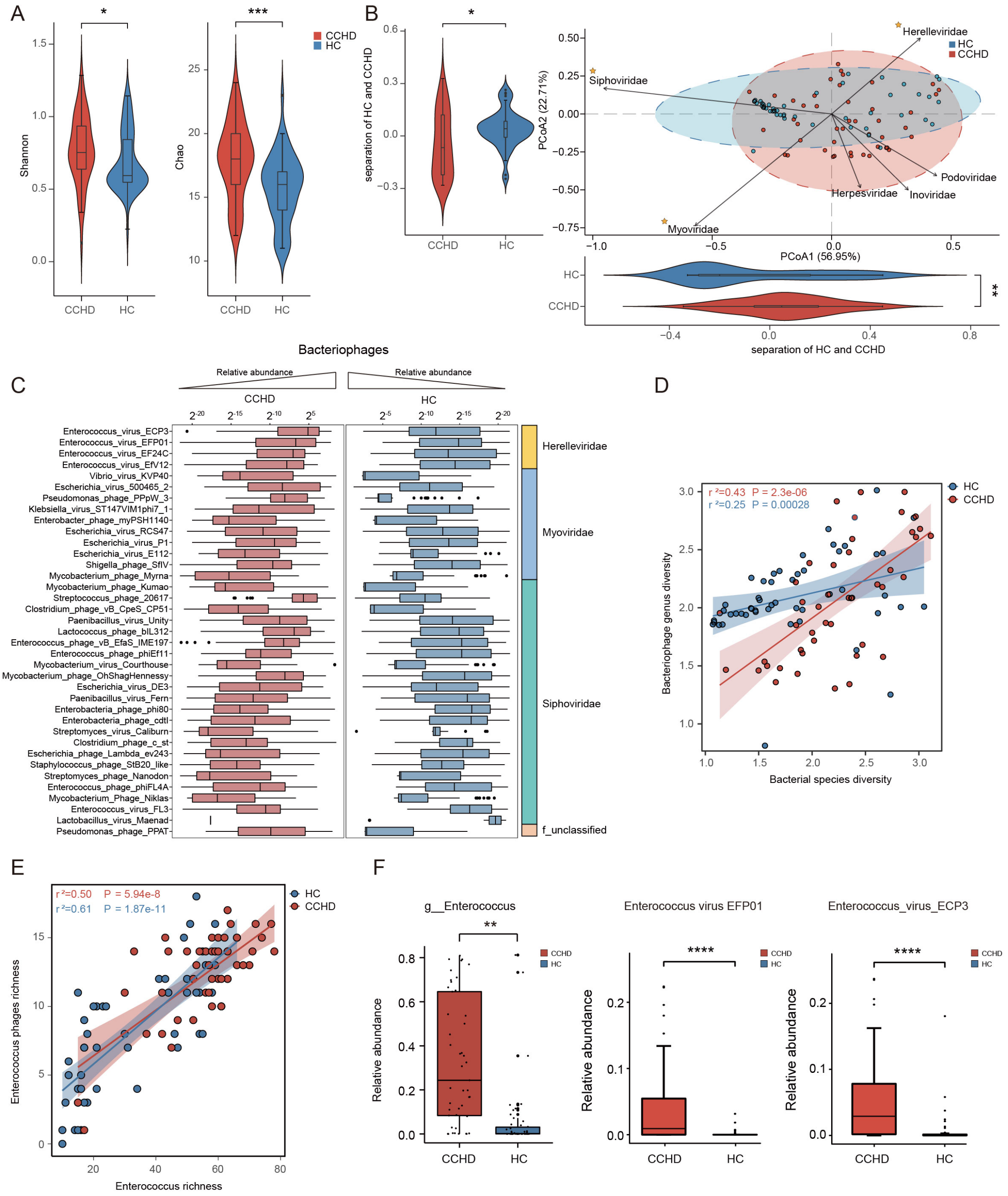

Figure S2. Gut virome alterations in CCHD associated with bacterial profile

(A) The comparison of gut viral α-diversity between CCHD and HC groups, as assessed by using Shannon and Chao indexes at the family level (Wilcoxon rank sum test).

(B) PCoA of the Bray–Curtis distances based on viral composition at the family level reveals significant difference between CCHD and HC groups (PERMANOVA, P=0.001). Viral taxa that significantly correlated with the PC-axes with Spearman's correlation coefficient > +0.35 or < −0.35 are graphed as contributors that drive the separation. The length of the arrow represents the degree of correlation to the PC-axes. The distribution and density of samples projected onto PC-axes are displayed in violin plots and assessed individually by using Wilcoxon rank sum test.

(C) Relative abundances of 37 viral species responsible for discriminating CCHD and HC groups (top 50 most abundant taxa with LDA score > 2.50). The family taxonomy of each species is shown on the right.

(D) Scatter plot shows positive associations between viral and bacterial microbiome diversities in CCHD and HC groups. The x axis refers to the Shannon index at the species level of bacterial microbiome, and the y axis refers to the Shannon index at the genus level of

viral microbiome. The fitted linear regression line is shown, with standardized R^2 and P value (determined by linear regression analysis). Shaded area around regression line represents 95% confidence range.

(E) Scatter plot shows positive associations in community richness between *Enterococcus* phages and their bacterial hosts (genus *Enterococcus*) in CCHD and HC groups. The x axis refers to the Chao index of *Enterococcus* phages at the species level and the y axis refers to the Chao index of *Enterococcus* (bacterial host) at the genus level. The fitted linear regression line is shown, with standardized R^2 and P value (determined by linear regression analysis). Shaded area around regression line represents 95% confidence range.

(F) The relative abundance of genus *Enterococcus* and *Enterococcus* phages (EFP01 and ECP3) in CCHD and HC groups (Wilcoxon rank sum test).

$^{*}P < 0.05$; $^{**}P < 0.01$; $^{***}P < 0.001$; $^{****}P < 0.0001$; CCHD, critical congenital heart disease; HC, healthy control; PCoA, principal coordinates analysis.

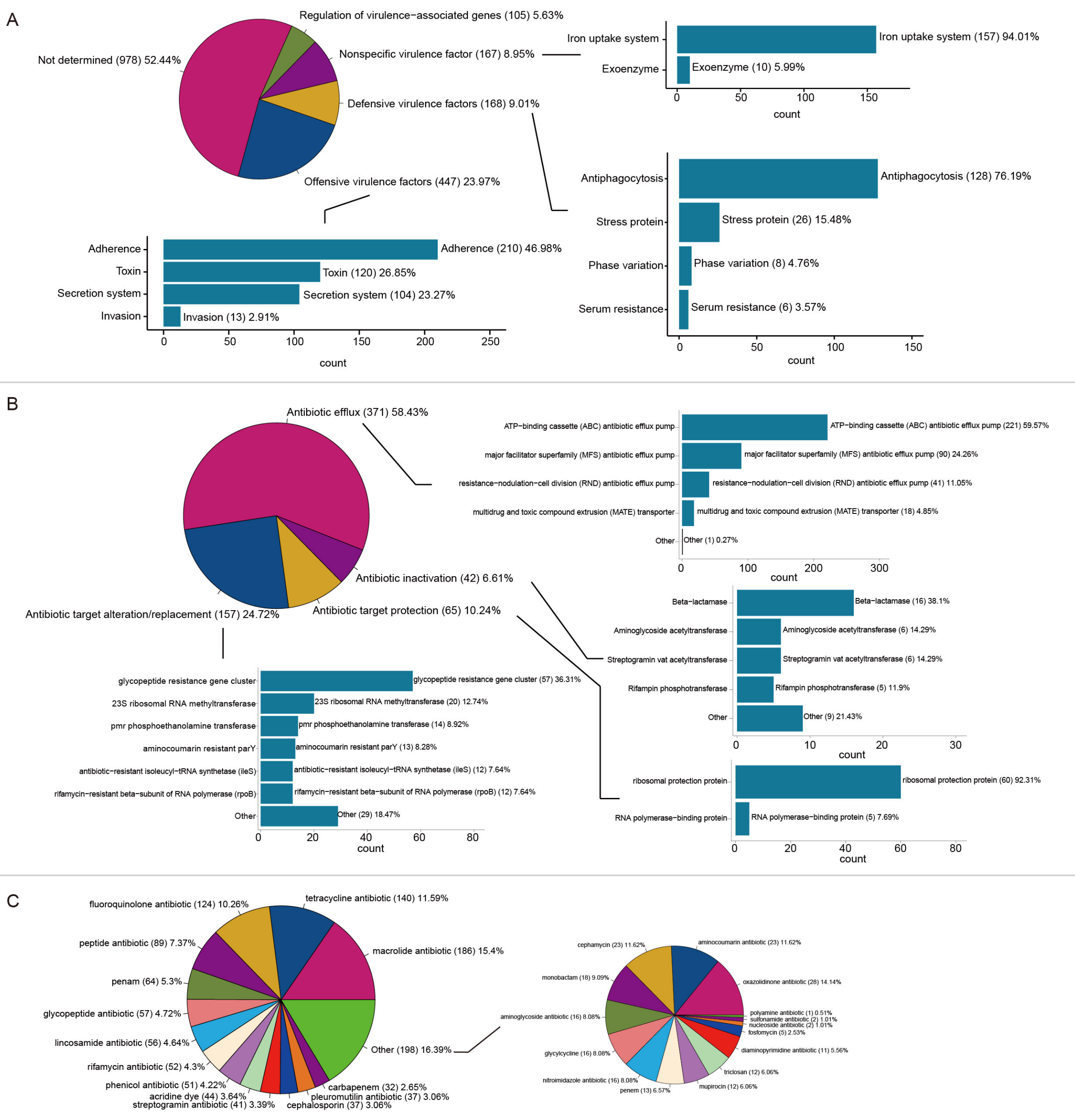

Figure S3. Functional genes associated with virulence factors and antibiotic resistance in *Siphoviridae* targeting *Enterococcus* in CCHD group.

(A) Distribution of virulence factor genes of *Siphoviridae* detected from *Enterococcus* contigs in CCHD group.

(B-C) Distribution of ARGs of *Siphoviridae* detected from *Enterococcus* contigs in CCHD group, classified by higher CARD categories **(B)** and drug classes **(C)**.

CCHD, critical congenital heart disease; ARG, antibiotic resistance gene.

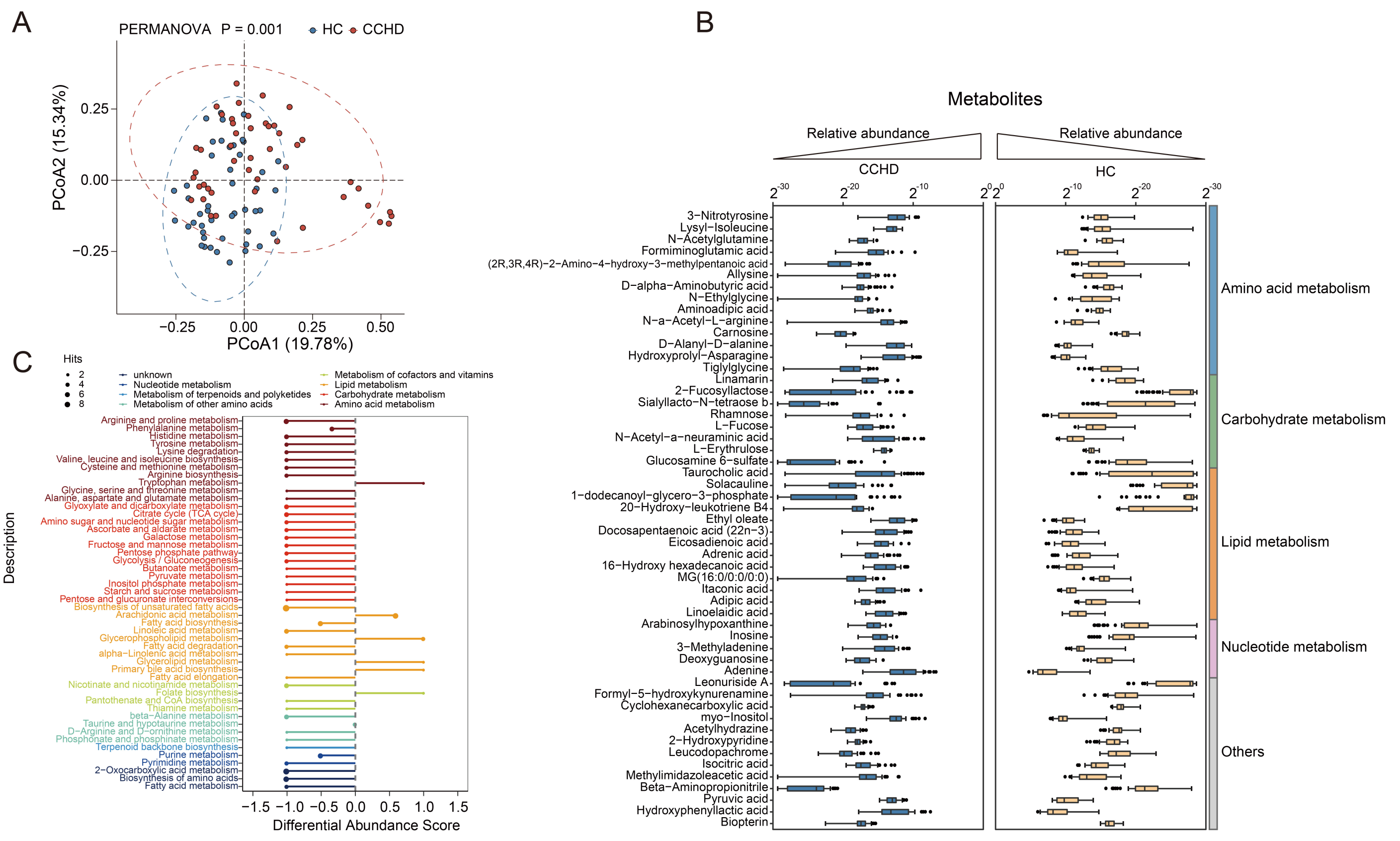

Figure S4. Differences in fecal metabolome between CCHD and HC

(A) PCoA based on the relative abundance of fecal metabolites reveals significant differences in the metabolomic profile between CCHD and HC groups (PERMANOVA). Dashed lined ellipses indicate 95% confidence interval (CI) of datapoints.

(B) Relative abundances of fecal metabolites responsible for discriminating CCHD and HC groups. For plotting purpose, only 53 metabolites with VIP score > 1.50 are shown. The associated metabolic pathways are shown on the right column.

(C) Pathway-based differential abundance (DA) analysis reveals differences in metabolic traits between CCHD and HC groups. A DA score > 0 indicates that the detected metabolites involved in pathway increase in CCHD relative to HC, whereas a DA score < 0 indicates that the detected metabolites involved in the pathway decrease in CCHD compared to HC. PCoA, principal coordinates analysis; CCHD, critical congenital heart disease; HC, healthy control.

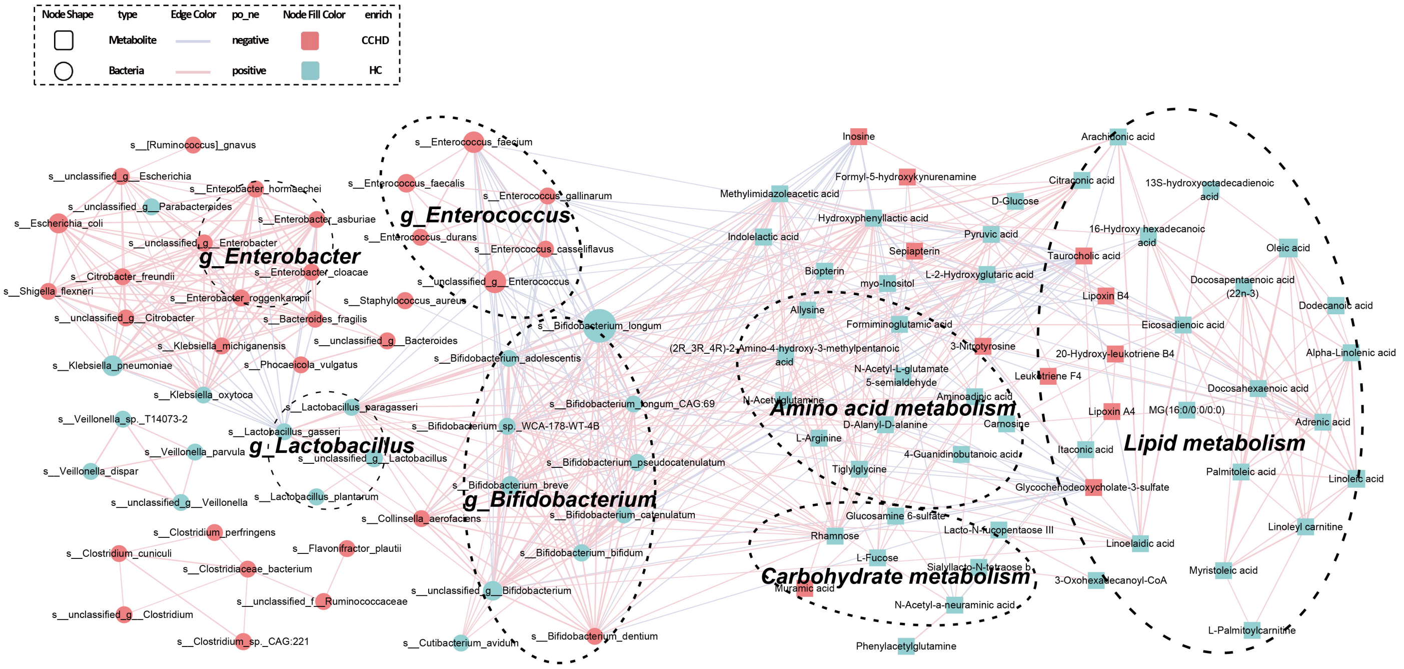

Figure S5. A co-occurrence network deduced from the differential bacterial species and fecal metabolites identified in CCHD *versus* HC

Correlations between variables are determined by Spearman's rank correlation. For plotting purpose, only significant correlations with correlation coefficients > +0.6 (light red edges) or < −0.6 (light blue edges) are graphed. The edge thickness indicates range of P value (P < 0.05). The size of each node is proportional to the mean relative abundance, and colored according to differential abundance results (light red nodes, variables enriched in CCHD; light blue nodes, variables enriched in HC). For bacterial species, four co-varying clusters are generated and annotated with genus taxonomy. The fecal metabolites are also clustered and labeled corresponding to the associated metabolic pathways. CCHD, critical congenital heart disease; HC, healthy control.

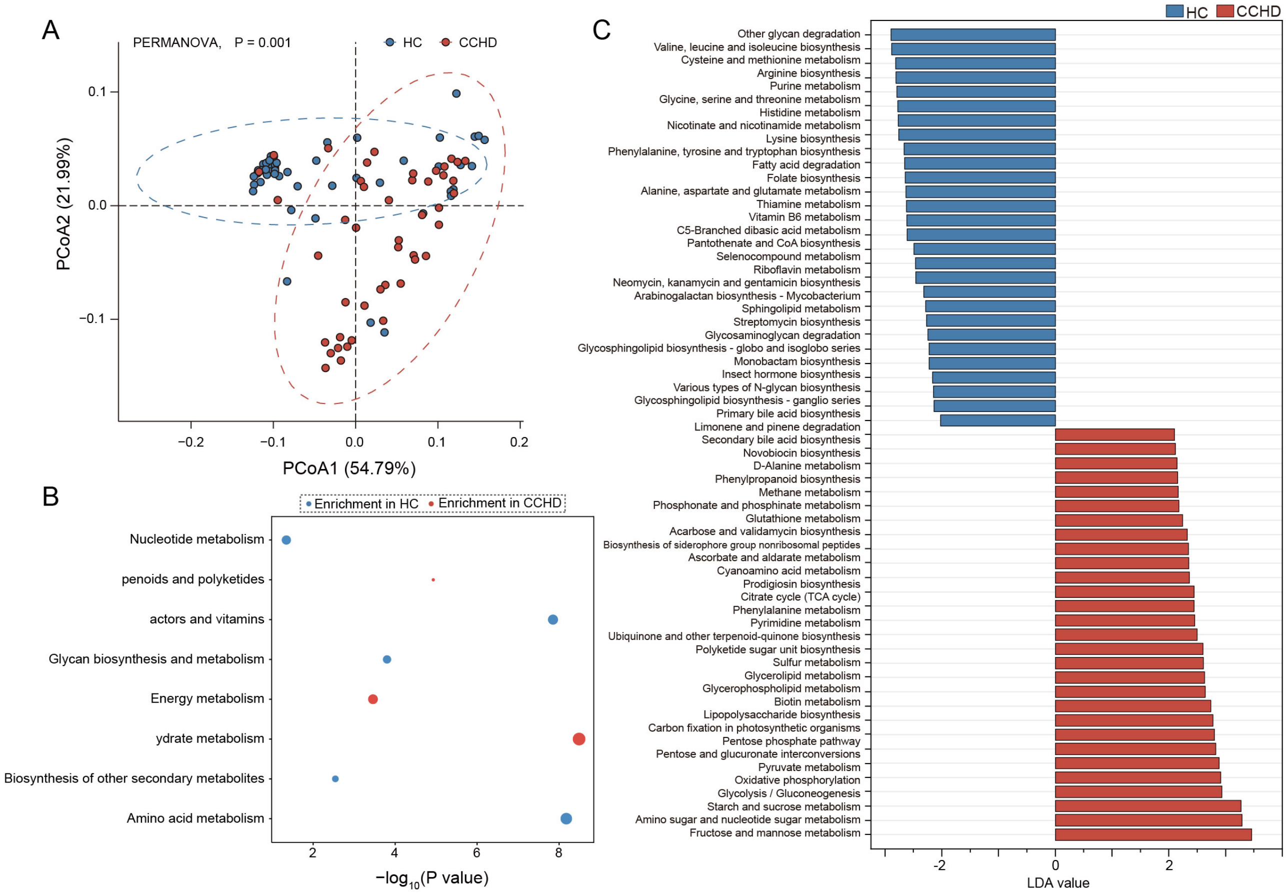

Figure S6. Alterations in microbial functionality between CCHD and HC

(A) PCoA based on the relative abundance of KEGG orthology groups reveals significant difference in microbial functionality between CCHD and HC groups (PERMANOVA). Dashed lined ellipses indicate 95% confidence interval (CI) of datapoints.

(B) Metabolic pathways (KEGG level 2) enriched by differential microbial genes in CCHD or HC. Abscissa variations indicate levels of significance (log10-transformed), and the size of nodes indicates the fold change.

(C) LDA showing KEGG metabolic pathways (level 3) responsible for discriminating the CCHD and HC groups (identified on the basis of LDA score > 2.0).

CCHD, critical congenital heart disease; HC, healthy control; PCoA, principal coordinates analysis; LDA, linear discriminant analysis.

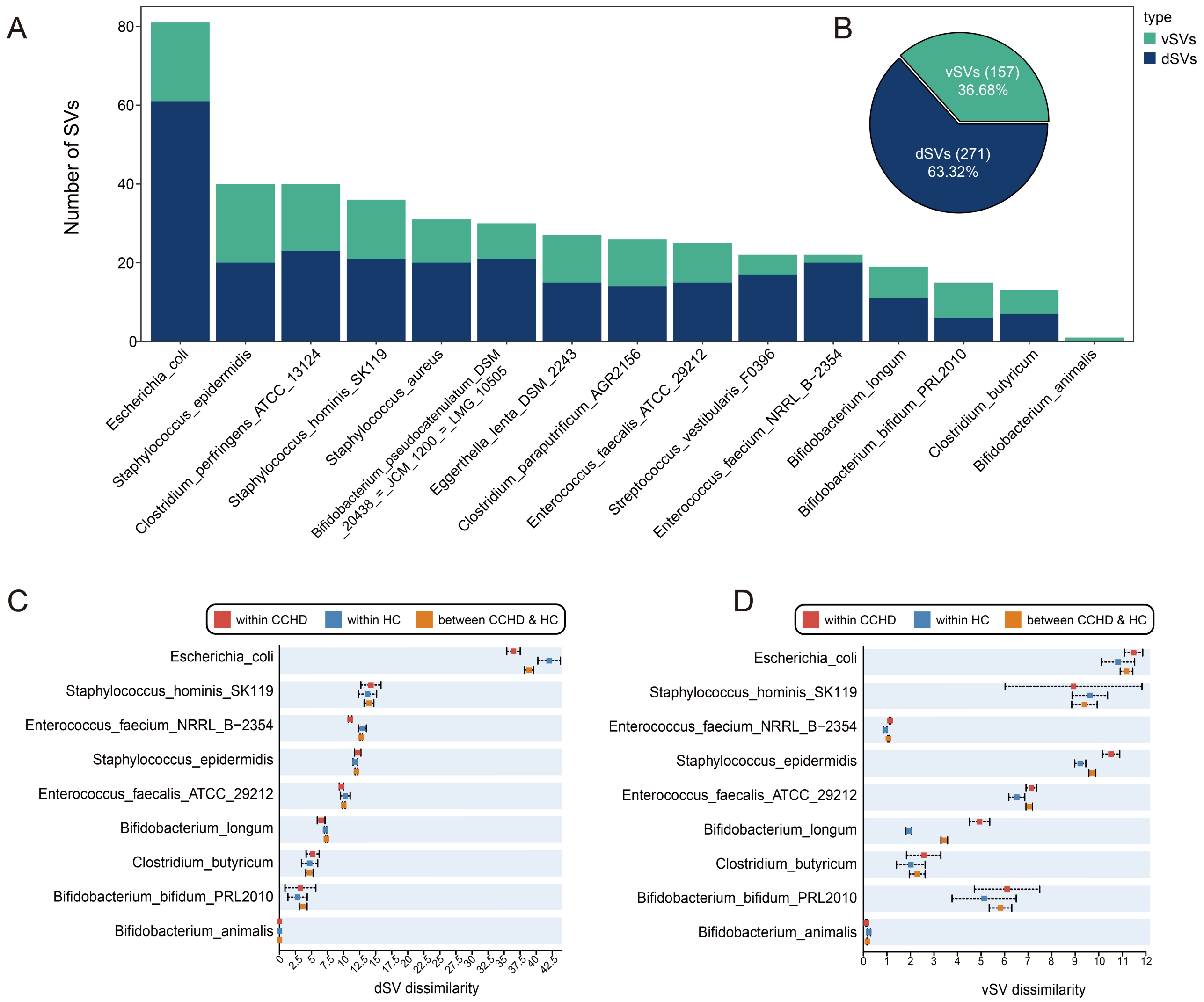

Figure S7. Overview of structural variation profile in gut microbiome in the study cohort

(A) Number of structural variants (SVs) of each bacterial species.

(B) Total number and distribution of variable SVs (vSVs) and deletion SVs (dSVs).

(C-D) Dissimilarity in the dSVs (C) and vSVs (D) of multiple bacterial species (red, dissimilarity within CCHD patients; blue, dissimilarity within HCs; orange, dissimilarity between CCHD and HC groups). Boxes and whiskers indicate median and 95% confidence interval (CI) of the data. CCHD, critical congenital heart disease; HC, healthy control.

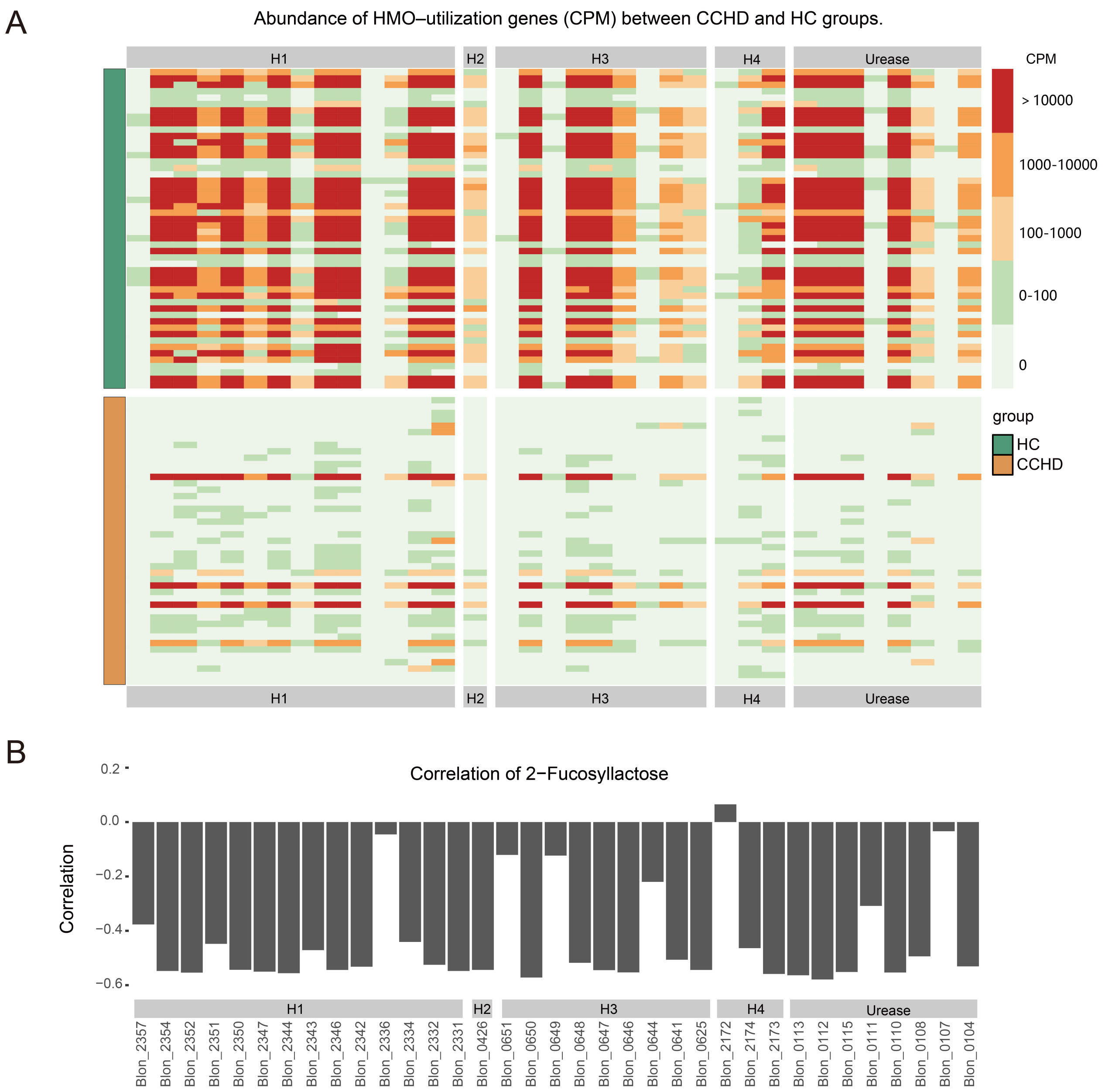

Figure S8 The abundance of human milk oligosaccharide (HMO)-utilization genes associated with fecal level of 2-fucosyllactose

(A) Heatmap shows abundance of the indicated HMO-utilization genes (columns) in the gut microbiome of CCHD and HC groups (horizontal groups).

(B) Spearman's correlation coefficients between the fecal level of 2-fucosyllactose and abundance of HMO-utilization genes..

CCHD, critical congenital heart disease; HC, healthy control; CPM, counts per million.

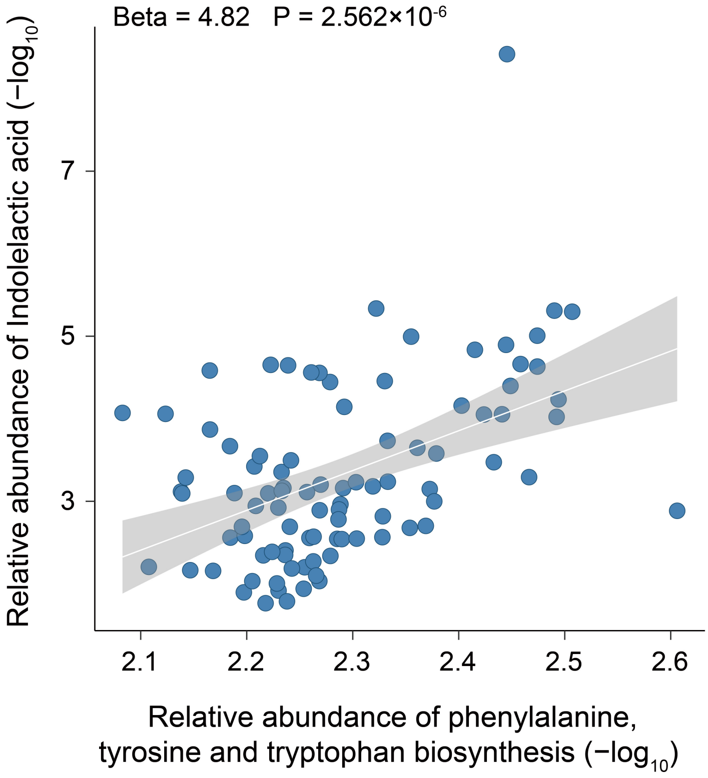

Figure S9. Association between microbial phenylalanine, tyrosine and tryptophan biosynthesis pathway and indolelactic acid

Scatter plots shows positive association between microbial phenylalanine, tyrosine and tryptophan biosynthesis pathway and indolelactic acid. The fitted linear regression line is shown, with standardized beta and P value.

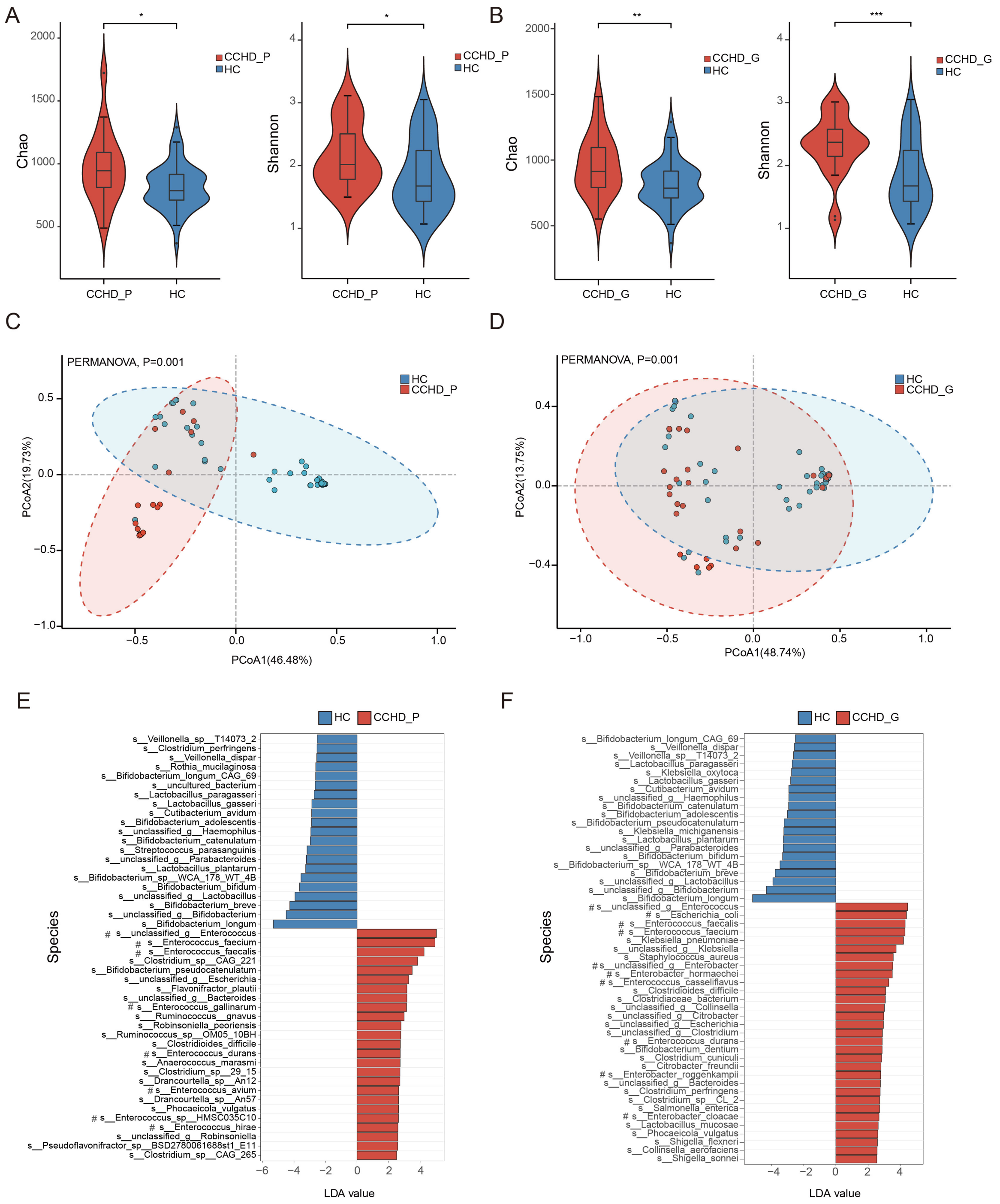

Figure S10. Differences in gut bacterial composition in pairwise comparisons of CCHD-P *versus* HC and CCHD-G *versus* HC

(A-B) The gut bacterial α-diversity in pairwise comparisons of CCHD-P *versus* HC **(A)** and CCHD-G *versus* HC **(B)**, as assessed by Chao and Shannon indexes at the species level (Wilcoxon rank sum test).

(C-D) PCoA of the Bray–Curtis distances based on bacterial composition at the species level reveals significant differences in pairwise comparisons of CCHD-P *versus* HC **(C)** and CCHD-G *versus* HC **(D)**. Statistical significance is tested by PERMANOVA.

(E-F) The discriminative bacterial species identified in pairwise comparisons of CCHD-P *versus* HC **(E)** and CCHD-G *versus* HC **(F)**. Bacterial taxa with LDA scores > 2.50 are shown. The Enterococcus and Enterobacter species enriched in CCHD-G and CCHD-P compared to HC are marked by "#".

*P < 0.05; **P < 0.01; ***P < 0.001; CCHD-P, CCHD patients with poor surgical prognosis; CCHD-G, CCHD patients with good surgical prognosis; PCoA, principal coordinates analysis; LDA, linear discriminant analysis.

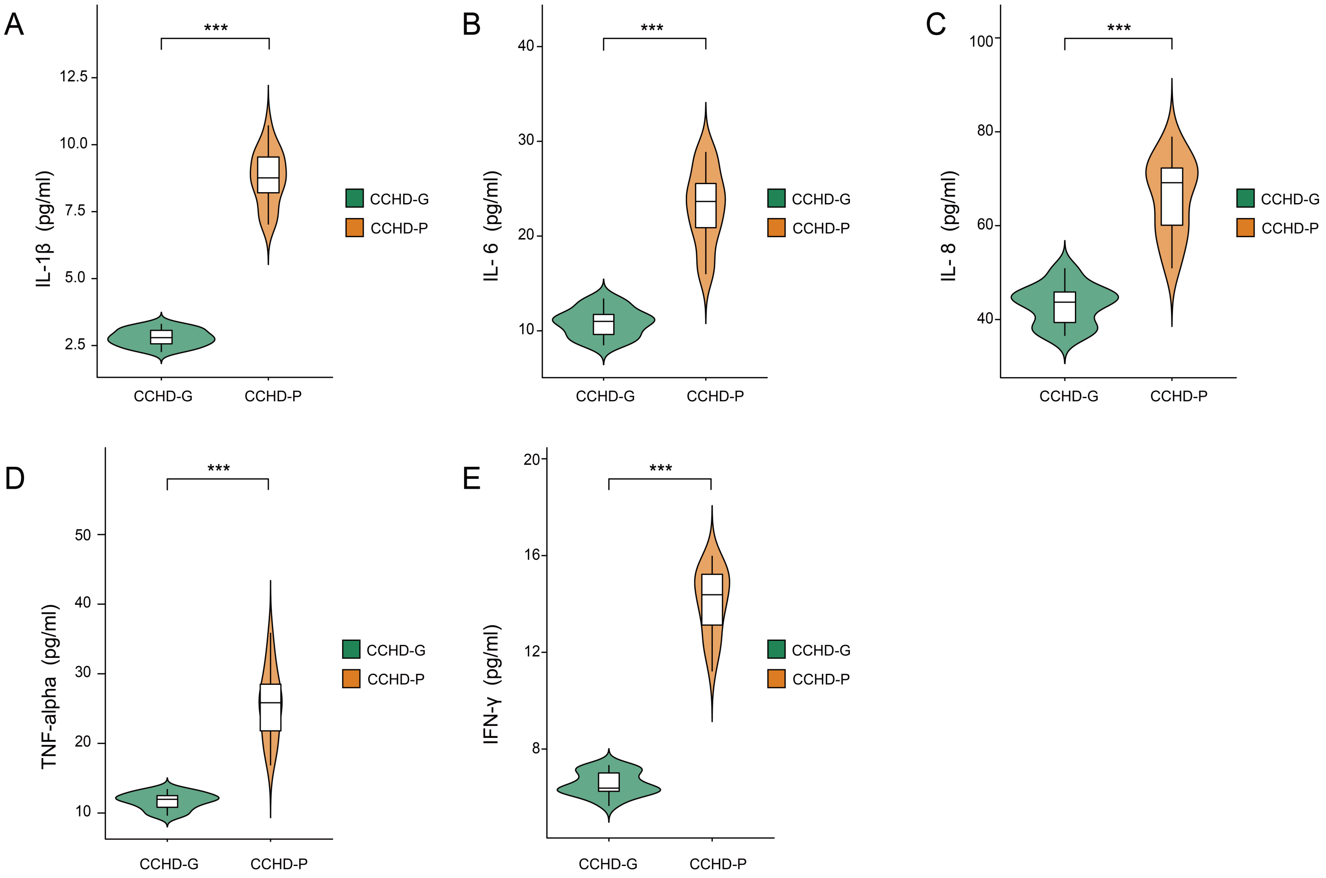

Figure S11. Comparison of serum inflammatory biomarkers between CCHD-G and CCHD-P subgroups. (A) IL-1 beta; (B) IL-6; (C) IL-8; (D) TNF-alpha; (E) IFN-γ.

*** P < 0.001; Mann–Whitney U-test. CCHD-G, CCHD patients with good surgical prognosis; CCHD-P, CCHD patients with poor surgical prognosis; IL, interleukin; TNF-alpha, tumor necrosis factor-alpha; IFN-γ, interferon-γ.

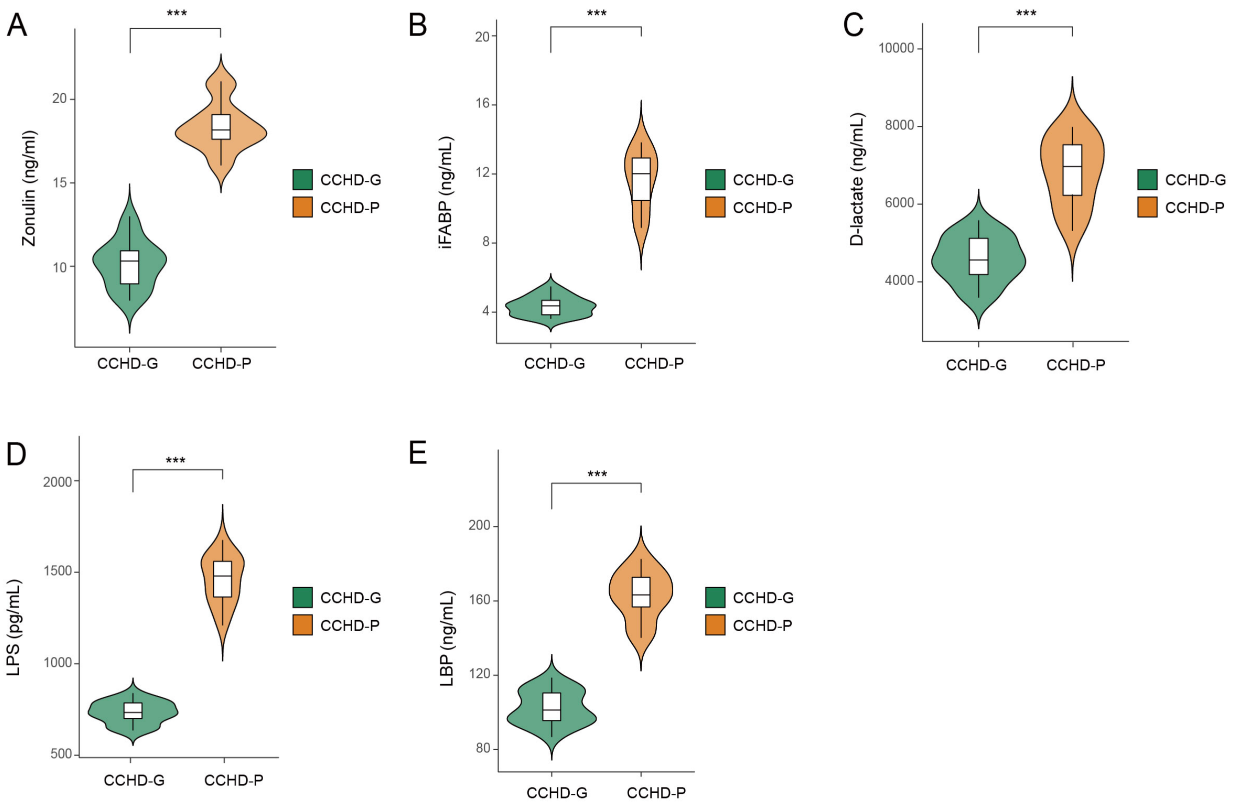

Figure S12. Comparison of intestinal permeability biomarkers between CCHD-G and CCHD-P subgroups. (A) Zonulin; (B) iFABP; (C) D-lactate; (D) LPS; (E) LBP.

*** P < 0.001; Mann–Whitney U-test. iFABP, intestinal fatty acid binding protein; LPS, lipopolysaccharide; LBP, lipopolysaccharide binding protein; CCHD-G, CCHD patients with good surgical prognosis; CCHD-P, CCHD patients with poor surgical prognosis.

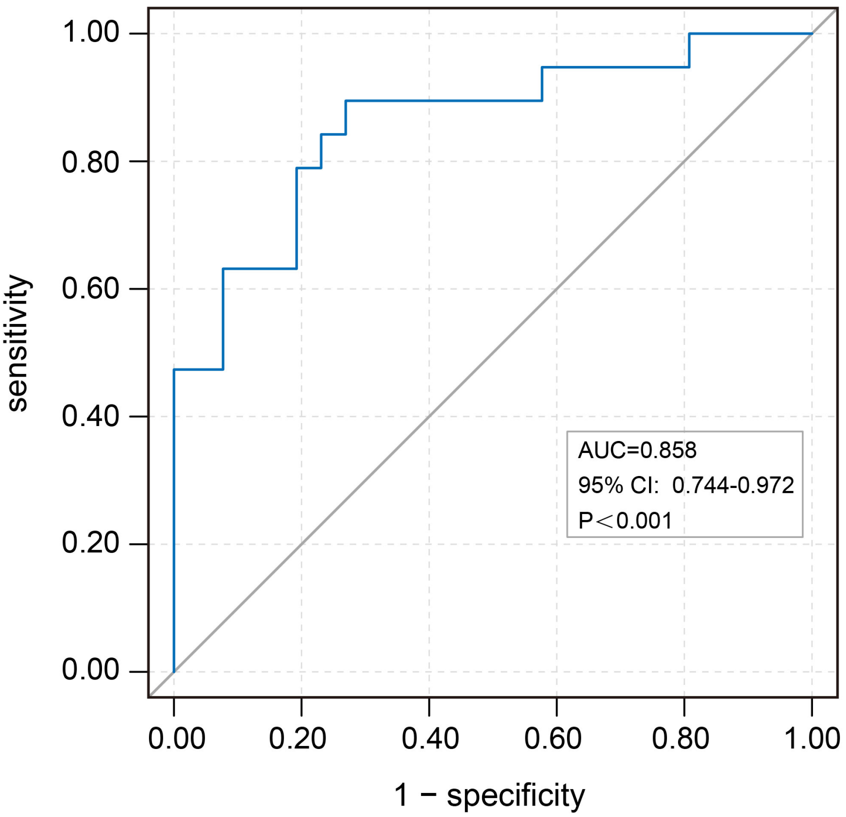

Figure S13. ROC curve showing discrimination accuracy of the predictive model for prognostic stratification in the study cohort

Area under the curve (AUC)=0.858 (95% CI: 0.744–0.972). The predictive model was constructed based on multivariable logistic regression analysis and comprised of *Enterococcus faecium* abundance, cardiopulmonary bypass time, and intraoperative infusion volume.

ROC, receiver-operating characteristic.

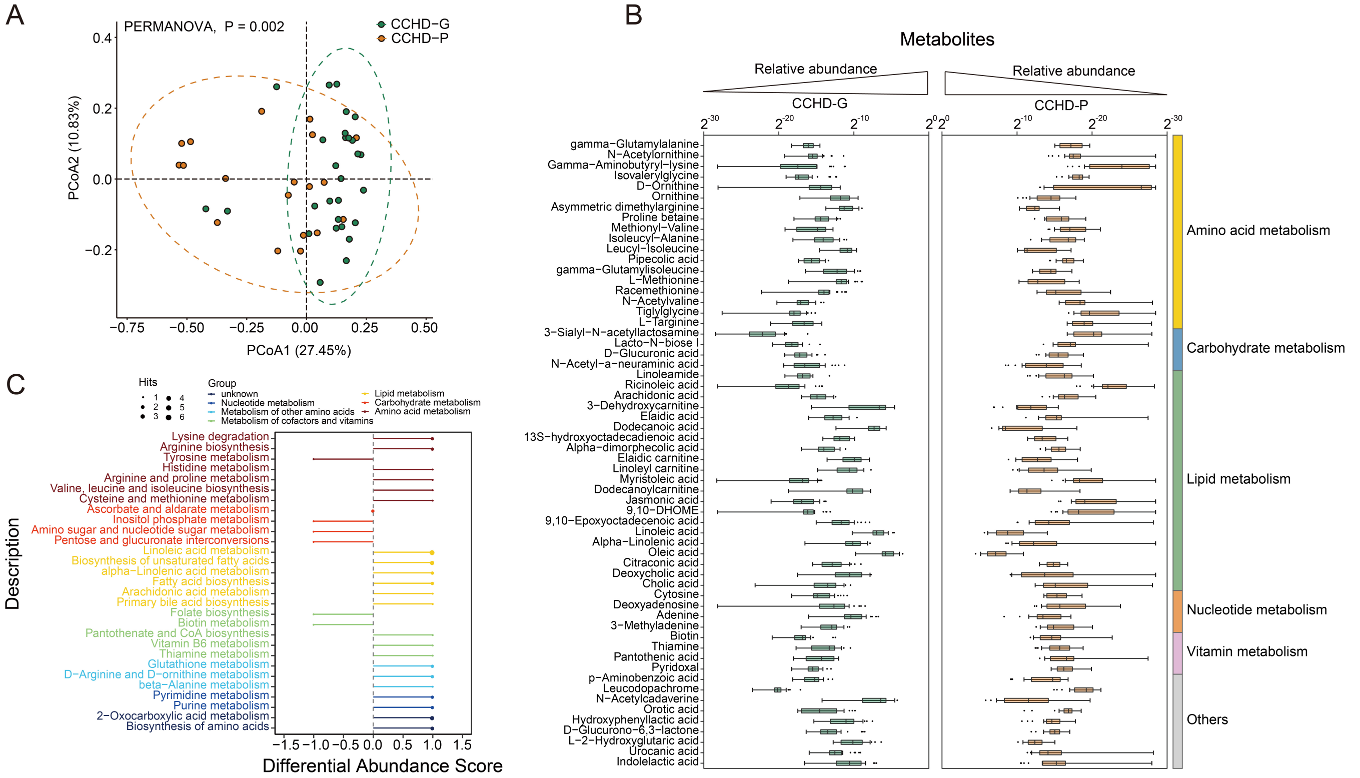

Figure S14. Differences in fecal metabolome between CCHD-P and CCHD-G

(A) PCoA based on the relative abundance of fecal metabolites reveals significant difference in metabolomic profile between CCHD-P and CCHD-G subgroups (PERMANOVA). Dashed lined ellipses indicate 95% confidence interval (CI) of datapoints.

(B) Relative abundances of 60 fecal metabolites responsible for discriminating CCHD-P and CHD-G groups (VIP score > 1.0). The associated metabolic pathways are shown on the right column.

(C) Pathway-based differential abundance (DA) analysis reveals differences in metabolic traits between CCHD-P and CCHD-G subgroups. A DA score < 0 indicates that the detected metabolites involved in pathway increase in CCHD-P relative to CCHD-G, whereas a DA score > 0 indicates that the detected metabolites involved in the pathway decrease in CCHD-P compared to CCHD-G.

CCHD-G, CCHD patients with good surgical prognosis; CCHD-P, CCHD patients with poor surgical prognosis; PCoA, principal coordinates analysis; VIP, variable importance in the projection.

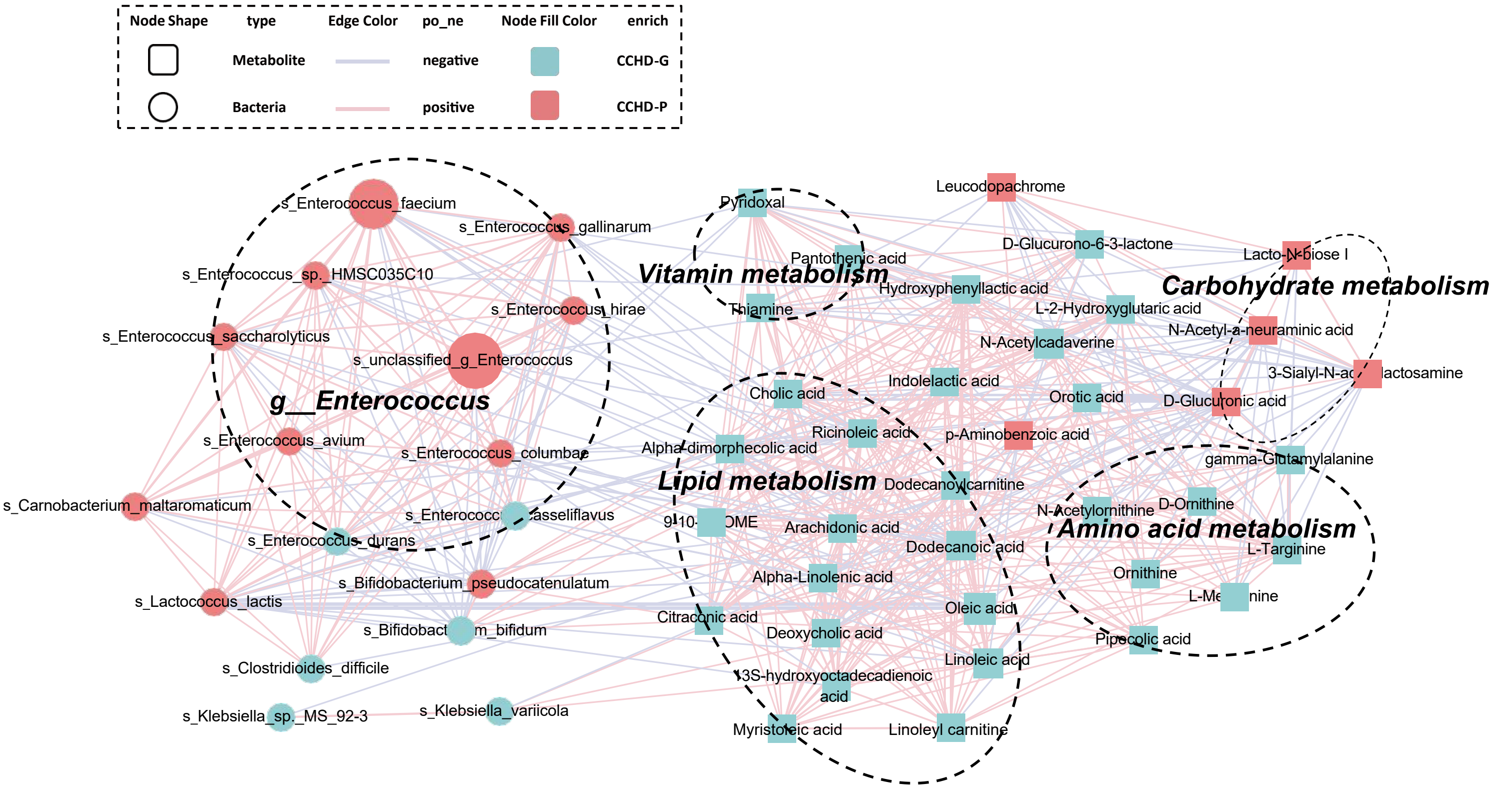

Figure S15. A co-occurrence network deduced from the differential bacterial species and fecal metabolites identified in CCHD-P *versus* CCHD-G

Correlations between variables are determined by Spearman's rank correlation. For plotting purpose, only significant correlations with correlation coefficients > +0.4 (light red edges) or < −0.4 (light blue edges) are graphed. The edge thickness indicates range of P value (P < 0.05). The size of each node is proportional to the mean relative abundance, and colored according to differential abundance results (light red nodes, variables enriched in CCHD-P; light blue nodes, variables enriched in CCHD-G). For bacterial species, a covarying cluster comprised of 8 *Enterococcus* species enriched in CCHD-P is generated and annotated with genus taxonomy. The fecal metabolites are also clustered and labeled corresponding to the associated metabolic pathways. CCHD-G, CCHD patients with good surgical prognosis; CCHD-P, CCHD patients with poor surgical prognosis.

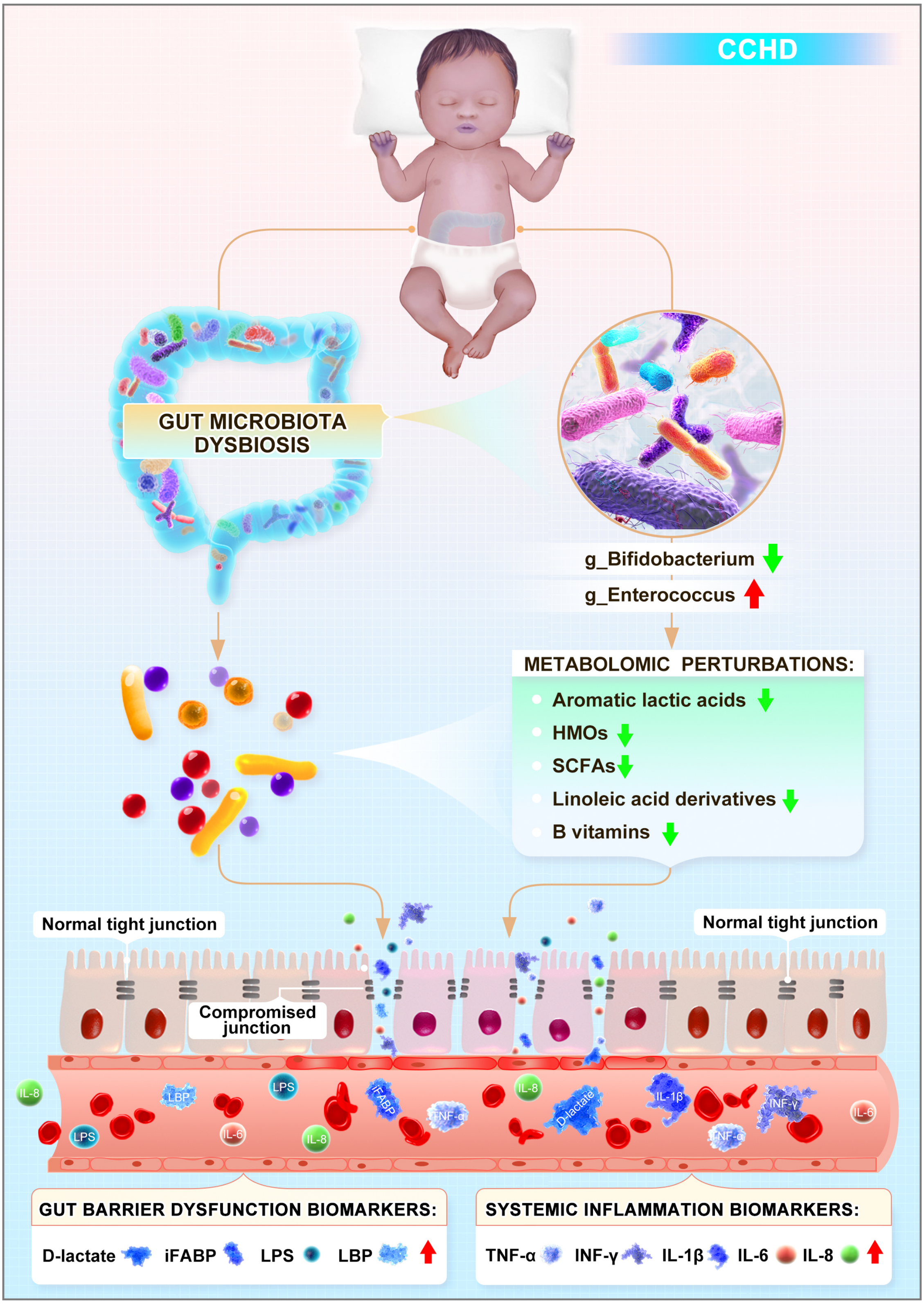

Figure S16. Conceptual diagram of potential mechanisms linking the gut microbiome, microbial metabolites and host pathological phenotypes including gut barrier impairment and systemic inflammatory response.

Based on the integrated correlation analysis and mediation analysis in the present study, we revealed that gut microbial perturbations in neonates with CCHD, characterized by the overgrowth of *Enterococcus* and depletion of *Bifidobacterium*, can affect the metabolism of aromatic lactic acids, HMOs, SCFAs, linoleic acid derivatives and B vitamins, thereby mediating gut barrier impairment and systemic inflammatory response. CCHD, critical congenital heart disease; HMOs, human milk oligosaccharides; SCFAs, short chain fatty acids.

REFERENCES

[1] Olney RS, Ailes EC, Sontag MK. Detection of critical congenital heart defects: Review of contributions from prenatal and newborn screening. Semin Perinatol 2015;39(3):230-7.

[2] Wu X, Luo Q, Su Z, Li Y, Wang H, Yuan S, et al. Prognostic Value of Preoperative Absolute Lymphocyte Count in Children With Tetralogy of Fallot. J Am Heart Assoc 2021;10(11):e019098.

[3] Yang J, Zheng P, Li Y, Wu J, Tan X, Zhou J, et al. Landscapes of bacterial and metabolic signatures and their interaction in major depressive disorders. Sci Adv 2020;6(49).

[4] Chen S, Zhou Y, Chen Y, Gu J. fastp: an ultra-fast all-in-one FASTQ preprocessor. Bioinformatics 2018;34(17):i884-i90.

[5] Li H, Durbin R. Fast and accurate short read alignment with Burrows-Wheeler transform. Bioinformatics 2009;25(14):1754-60.

[6] Li D, Liu CM, Luo R, Sadakane K, Lam TW. MEGAHIT: an ultra-fast single-node solution for large and complex metagenomics assembly via succinct de Bruijn graph. Bioinformatics 2015;31(10):1674-6.

[7] Hyatt D, Chen GL, Locascio PF, Land ML, Larimer FW, Hauser LJ. Prodigal: prokaryotic gene recognition and translation initiation site identification. BMC Bioinformatics 2010;11:119.

[8] Fu L, Niu B, Zhu Z, Wu S, Li W. CD-HIT: accelerated for clustering the next-generation sequencing data. Bioinformatics 2012;28(23):3150-2.

[9] Li R, Li Y, Kristiansen K, Wang J. SOAP: short oligonucleotide alignment program. Bioinformatics 2008;24(5):713-4.

[10] Kanehisa M, Goto S. KEGG: kyoto encyclopedia of genes and genomes. Nucleic Acids Res 2000;28(1):27-30.

[11] Liu F, Smith AD, Solano-Aguilar G, Wang TTY, Pham Q, Beshah E, et al. Mechanistic insights into the attenuation of intestinal inflammation and modulation of the gut microbiome by krill oil using in vitro and in vivo models. Microbiome 2020;8(1):83.

[12] Seki D, Mayer M, Hausmann B, Pjevac P, Giordano V, Goeral K, et al. Aberrant gut-microbiota-immune-brain axis development in premature neonates with brain damage. Cell Host Microbe 2021;29(10):1558-72 e6.

[13] Fasano A. Zonulin and its regulation of intestinal barrier function: the biological door to inflammation, autoimmunity, and cancer. Physiol Rev 2011;91(1):151-75.

[14] Yao YM, Yu Y, Wu Y, Lu LR, Sheng ZY. Plasma D (-)-lactate as a new marker for diagnosis of acute intestinal injury following ischemia-reperfusion. World J Gastroenterol 1997;3(4):225-7.

[15] Sun XQ, Fu XB, Zhang R, Lu Y, Deng Q, Jiang XG, et al. Relationship between plasma D(-)-lactate and intestinal damage after severe injuries in rats. World J Gastroenterol 2001;7(4):555-8.

[16] Lau E, Marques C, Pestana D, Santoalha M, Carvalho D, Freitas P, et al. The role of I-FABP as a biomarker of intestinal barrier dysfunction driven by gut microbiota changes in obesity. Nutr Metab (Lond) 2016;13:31.

[17] Perez-Hernandez EG, Delgado-Coello B, Luna-Reyes I, Mas-Oliva J. New insights into lipopolysaccharide inactivation mechanisms in sepsis. Biomed Pharmacother 2021;141:111890.

[18] Vanuytsel T, Tack J, Farre R. The Role of Intestinal Permeability in Gastrointestinal Disorders and Current Methods of Evaluation. Front Nutr 2021;8:717925.

[19] Schumann RR, Leong SR, Flaggs GW, Gray PW, Wright SD, Mathison JC, et al. Structure and function of lipopolysaccharide binding protein. Science 1990;249(4975):1429-31.

[20] Zeevi D, Korem T, Godneva A, Bar N, Kurilshikov A, Lotan-Pompan M, et al. Structural variation in the gut microbiome associates with host health. Nature 2019;568(7750):43-8.

[21] Santos-Medellin C, Zinke LA, Ter Horst AM, Gelardi DL, Parikh SJ, Emerson JB. Viromes outperform total metagenomes in revealing the spatiotemporal patterns of agricultural soil viral communities. ISME J 2021;15(7):1956-70.

[22] Camarillo-Guerrero LF, Almeida A, Rangel-Pineros G, Finn RD, Lawley TD. Massive expansion of human gut bacteriophage diversity. Cell 2021;184(4):1098-109 e9.

[23] Nayfach S, Paez-Espino D, Call L, Low SJ, Sberro H, Ivanova NN, et al. Metagenomic compendium of 189,680 DNA viruses from the human gut microbiome. Nat Microbiol 2021;6(7):960-70.

[24] Paez-Espino D, Chen IA, Palaniappan K, Ratner A, Chu K, Szeto E, et al. IMG/VR: a database of cultured and uncultured DNA Viruses and retroviruses. Nucleic Acids Res 2017;45(D1):D457-D65.

[25] Pickett BE, Sadat EL, Zhang Y, Noronha JM, Squires RB, Hunt V, et al. ViPR: an open bioinformatics database and analysis resource for virology research. Nucleic Acids Res 2012;40(Database issue):D593-8.

[26] Fujimoto K, Kimura Y, Shimohigoshi M, Satoh T, Sato S, Tremmel G, et al. Metagenome Data on Intestinal Phage-Bacteria Associations Aids the Development of Phage Therapy against Pathobionts. Cell Host Microbe 2020;28(3):380-9 e9.

[27] Fujimoto K, Kimura Y, Allegretti JR, Yamamoto M, Zhang YZ, Katayama K, et al. Functional Restoration of Bacteriomes and Viromes by Fecal Microbiota Transplantation. Gastroenterology 2021;160(6):2089-102 e12.

[28] Roux S, Hallam SJ, Woyke T, Sullivan MB. Viral dark matter and virus-host interactions resolved from publicly available microbial genomes. Elife 2015;4.

[29] Hyatt D, LoCascio PF, Hauser LJ, Uberbacher EC. Gene and translation initiation site prediction in metagenomic sequences. Bioinformatics 2012;28(17):2223-30.

[30] Finn RD, Clements J, Eddy SR. HMMER web server: interactive sequence similarity searching. Nucleic Acids Res 2011;39(Web Server issue):W29-37.

[31] Finn RD, Coggill P, Eberhardt RY, Eddy SR, Mistry J, Mitchell AL, et al. The Pfam protein families database: towards a more sustainable future. Nucleic Acids Res 2016;44(D1):D279-85.

[32] Chen L, Zheng D, Liu B, Yang J, Jin Q. VFDB 2016: hierarchical and refined dataset for big data analysis--10 years on. Nucleic Acids Res 2016;44(D1):D694-7.

[33] Alcock BP, Raphenya AR, Lau TTY, Tsang KK, Bouchard M, Edalatmand A, et al. CARD 2020: antibiotic resistome surveillance with the comprehensive antibiotic resistance database. Nucleic Acids Res 2020;48(D1):D517-D25.

[34] Biswas A, Staals RH, Morales SE, Fineran PC, Brown CM. CRISPRDetect: A flexible algorithm to define CRISPR arrays. BMC Genomics 2016;17:356.

[35] Arumugam M, Raes J, Pelletier E, Le Paslier D, Yamada T, Mende DR, et al. Enterotypes of the human gut microbiome. Nature 2011;473(7346):174-80.
